# Supplementary material for: DG–CA3 circuitry mediates hippocampal representations of latent information
Source: Nat Commun. 2020 Jun 15;11:3026. doi: 10.1038/s41467-020-16825-1 (PMC7296021; doi:10.1038/s41467-020-16825-1)
Supplement: Supplementary file 1 — Supplementary Information [file 41467_2020_16825_MOESM1_ESM.pdf]

Supplementary information for:

DG-CA3 circuitry mediates hippocampal representations of latent information

Keinath, et al.

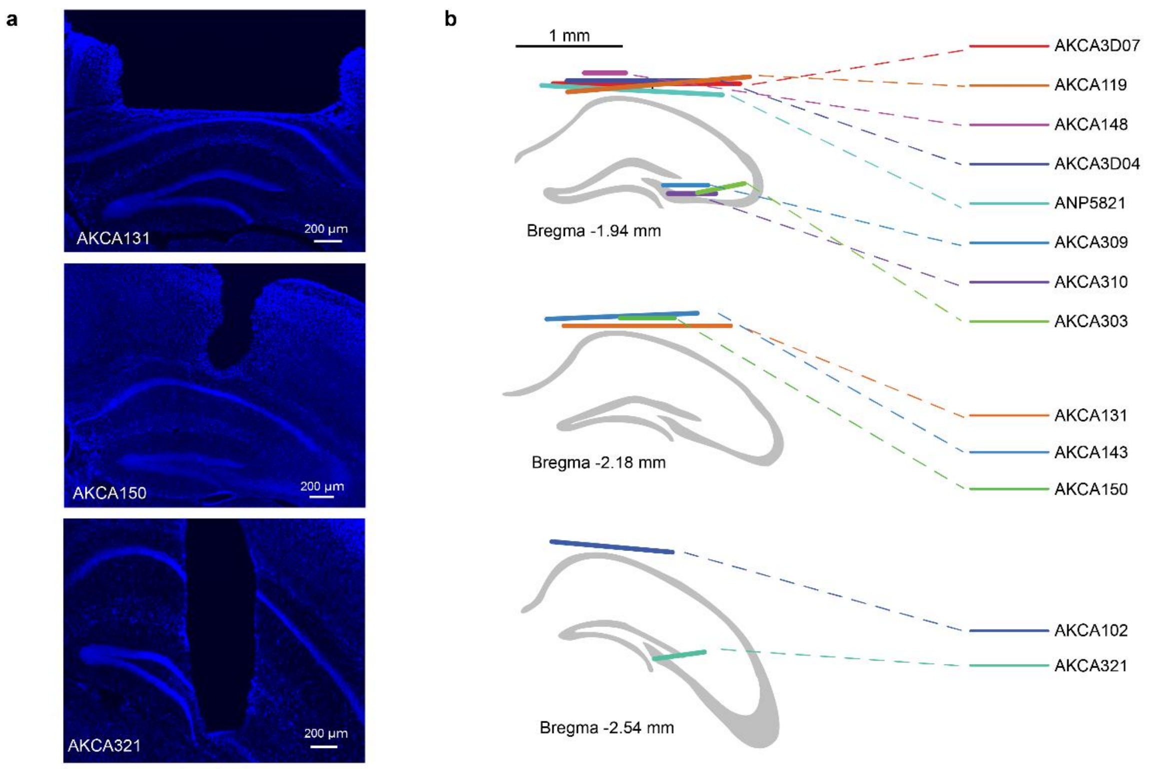

Supplementary Figure 1. Histological characterization of recording locations. a) Representative examples of 1.8 mm diameter GRIN lens placement in CA1 (top; one of seven mice for which localizing images were taken), 0.5 mm diameter relay lens placement in CA1 (middle; one of two mice for which localizing images were taken), and 0.5 mm diameter relay lens placement in CA3 (bottom; one of four mice for which localizing images were taken). b) Histologically-confirmed lens locations (AP determined from the slide with the clearest lens tract). The lens placement in a subset of 1.8mm diameter GRIN CA1 recorded animals were not verified after recording (AKCA133, AKCA142, AKCA110, and AKCA115). Bregma coordinates derived from[1].

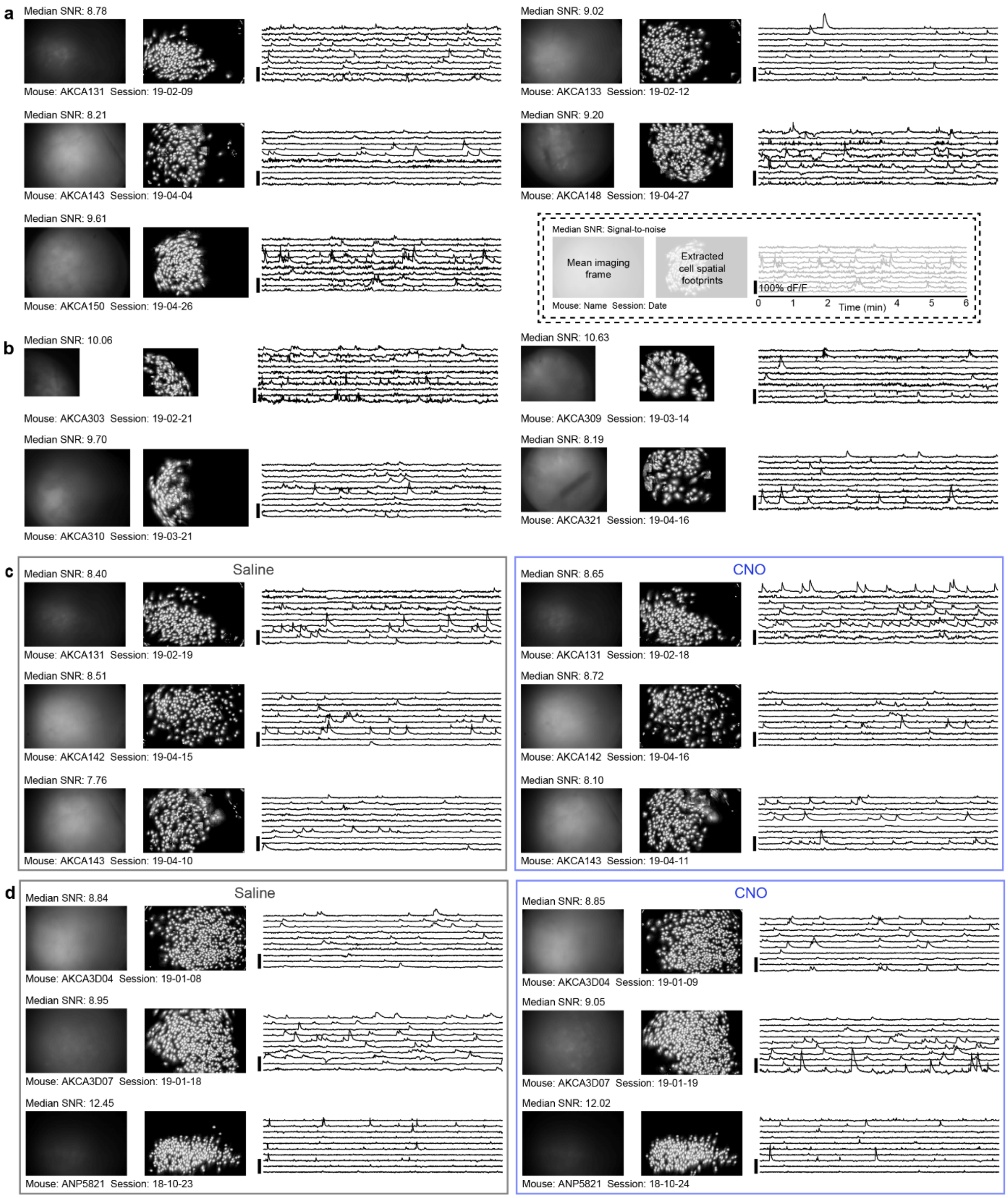

Supplementary Figure 2. Mean imaging frames, extracted cell spatial footprints, and dF/F cell traces for example sessions from each mouse. Traces are from 10 random cells from each session prior to spike estimation via deconvolution. a) One example session from each mouse included in the initial CA1 recording experiments. b) One example session from each mouse included in the CA3 recording experiments. c) Two examples (one under saline, one under CNO) from consecutive sessions for each control mouse without hm4di from the trisynaptic inhibition experiment. d) Two examples (one under saline, one under CNO) from

consecutive sessions for each experimental mouse expressing hm4di from the trisynaptic inhibition experiment.

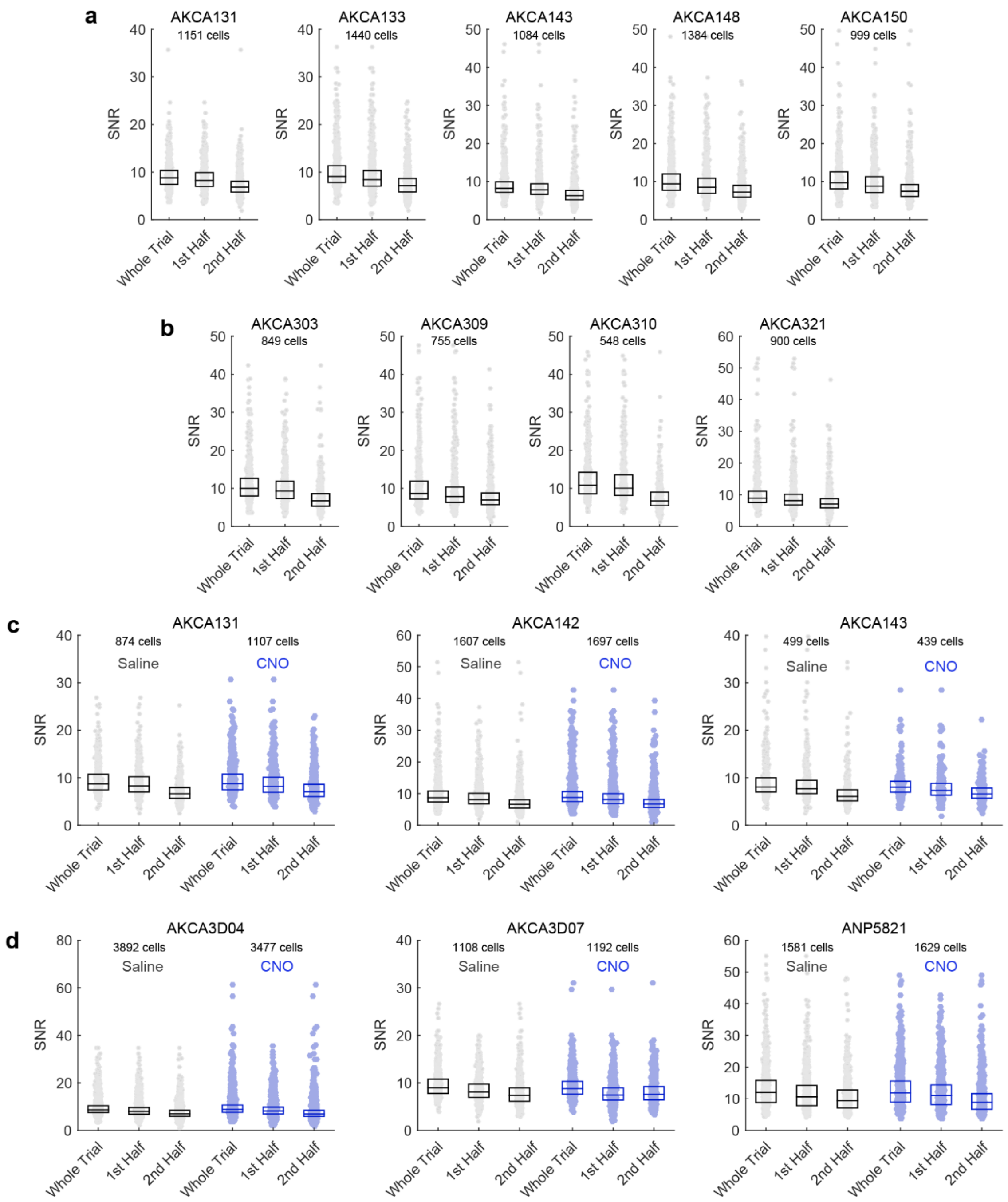

Supplementary Figure 3. Signal-to-noise (SNR) for each mouse in each experiment. SNR for all cells computed to spike estimation via deconvolution, collapsed across all recording sessions for each mouse, computed separately across the entire session and each session half. Box and line indicate interquartile range and median, respectively. a) dF/F SNR for all mice in the initial CA1 recording experiment. b) dF/F SNR for all mice in the CA3 recording experiment. c) dF/F SNR for all control mice without hm4di in the trisynaptic inhibition recording experiment, separated by saline and CNO sessions. d) dF/F SNR for all mice with hm4di in the trisynaptic inhibition recording experiment, separated by saline and CNO sessions. Data provided as Source Data file.

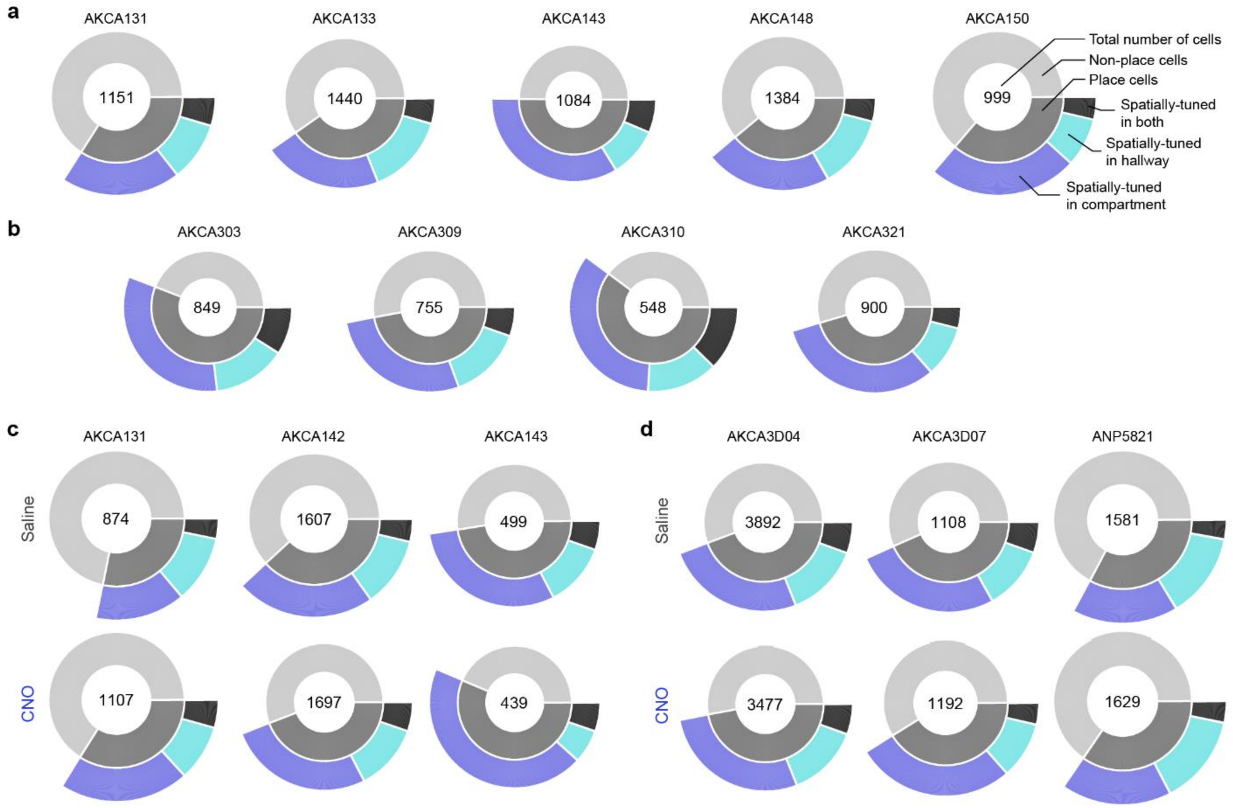

Supplementary Figure 4. Breakdown of cells meeting selection criteria for each mouse. Data collapsed across all recording sessions for each mouse. a) Breakdown of cells meeting selection criteria for all mice in the initial CA1 recording experiment. b) Breakdown of cells meeting selection criteria for all mice in the CA3 recording experiment. c) Breakdown of cells meeting selection criteria for all control mice without hm4di in the trisynaptic inhibition recording experiment, separated by saline and CNO sessions. d) Breakdown of cells meeting selection criteria for all mice with hm4di in the trisynaptic inhibition recording experiment, separated by saline and CNO sessions. Data provided as Source Data file.

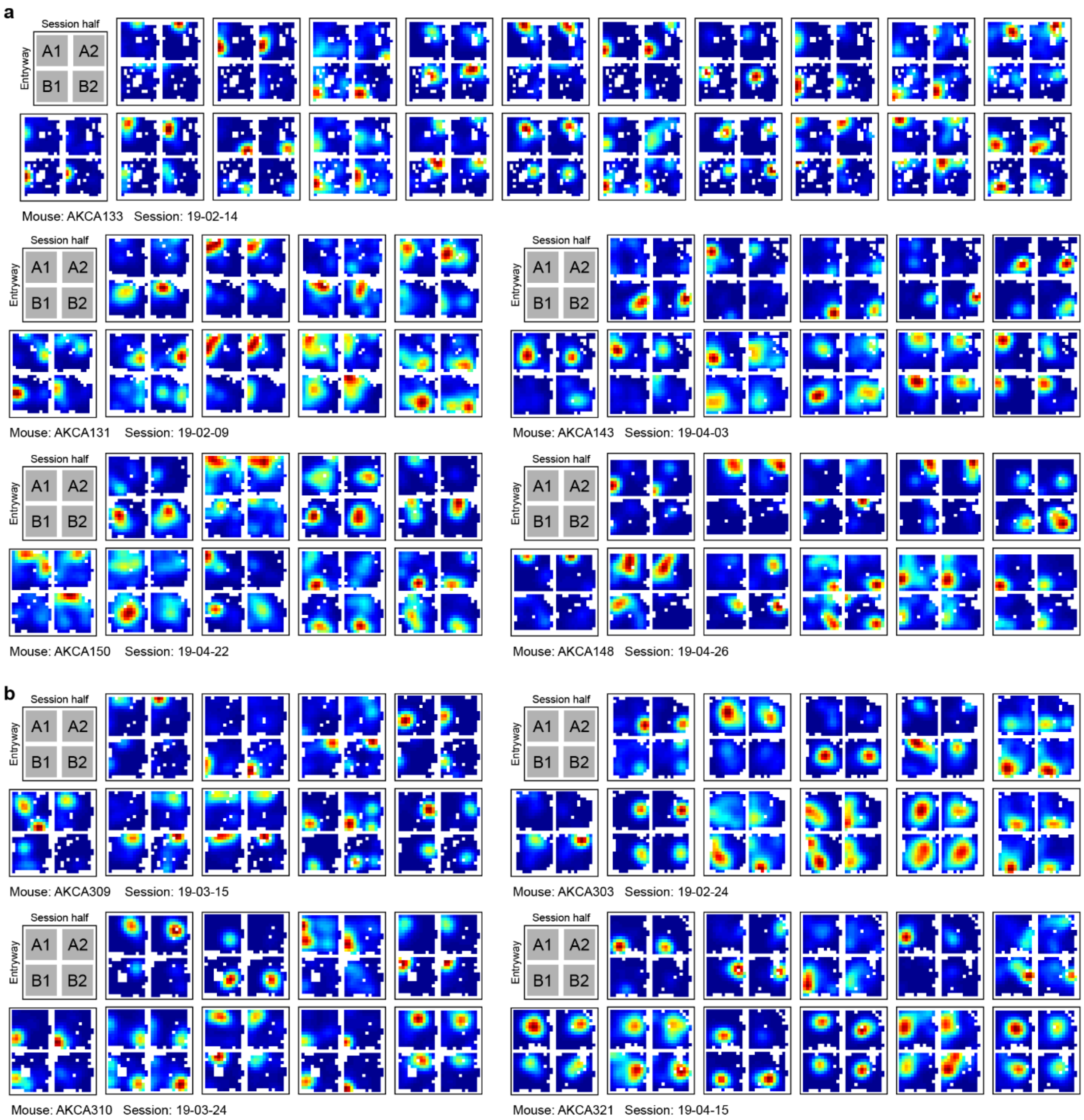

Supplementary Figure 5. Additional examples of CA1 and CA3 rate maps. Example rate maps from simultaneously recorded place cells for one session from each mouse when the data are divided by entryway and session half. Rate maps are normalized from zero (blue) to the peak (red) across all four maps. a) Initial CA1 recordings. b) CA3 recordings.

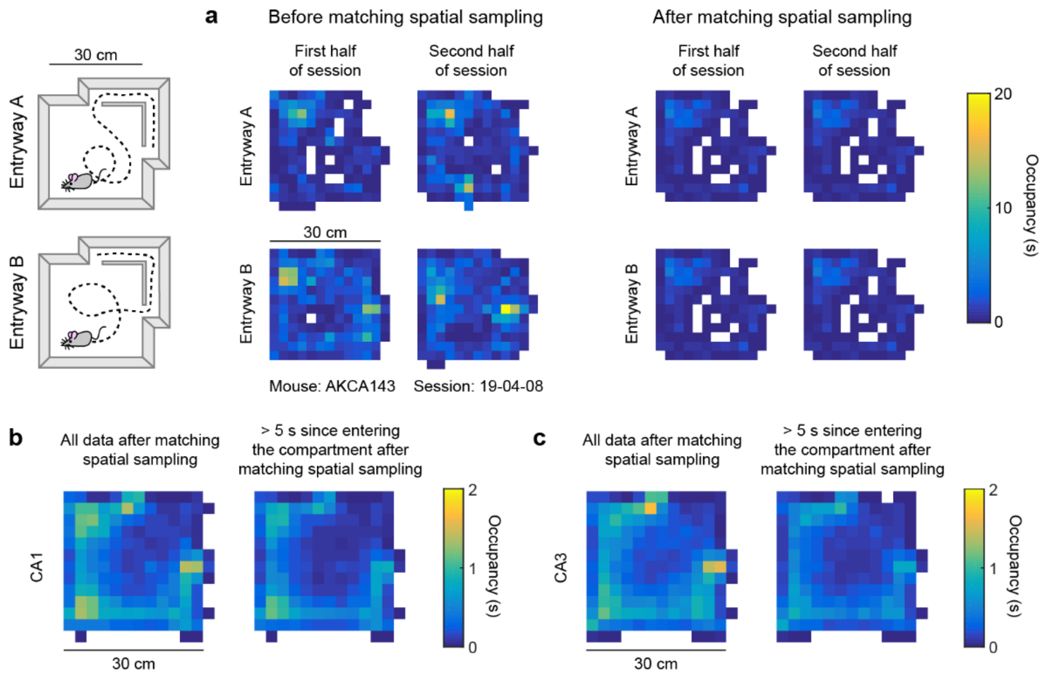

Supplementary Figure 6. Data were subsampled to match spatial sampling distributions across all conditions prior to all analyses. a) Biases in the sampling of spatial locations within the compartment may be correlated with the most recent entryway. To control for these possible biases, we subsampled the data to match the sampling distributions across all conditions prior to all analyses. b) Spatial sampling after matching spatial sampling distributions across all split-half comparisons for all data (left) and only data recorded after at least 5 s since the mouse entered the compartment (right) for the initial CA1 recording sessions. c) as in (b) except for CA3 recording sessions.

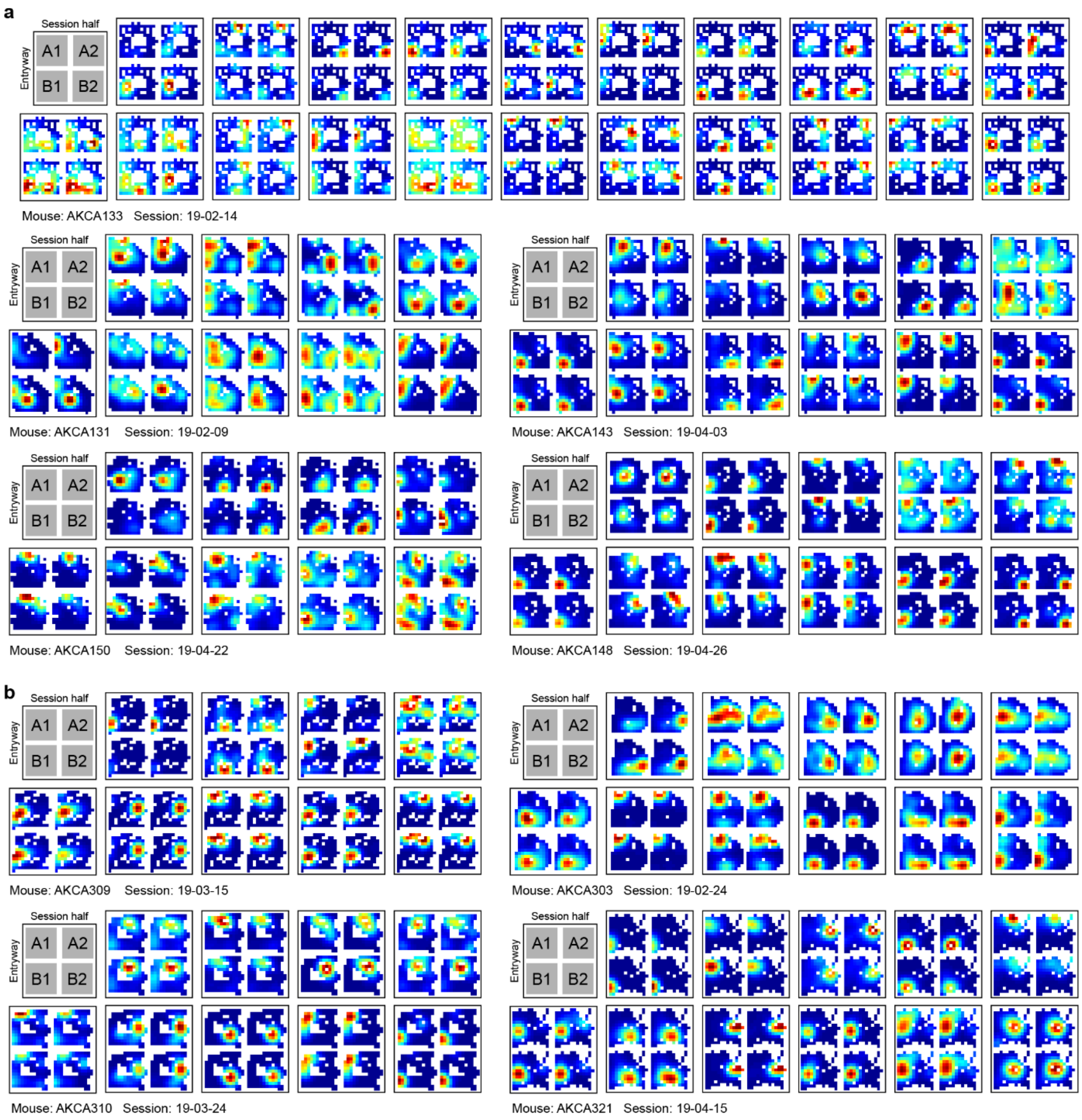

Supplementary Figure 7. Examples of CA1 and CA3 rate maps for one iteration of matching spatial sampling. Example rate maps from simultaneously recorded place cells for one session from each mouse when the data are divided by entryway and session half after matching spatial sampling across halves and entryways. Selected sessions as in Figure S4. Rate maps are normalized from zero (blue) to the peak (red) across all four maps. a) Initial CA1 recordings. b) CA3 recordings.

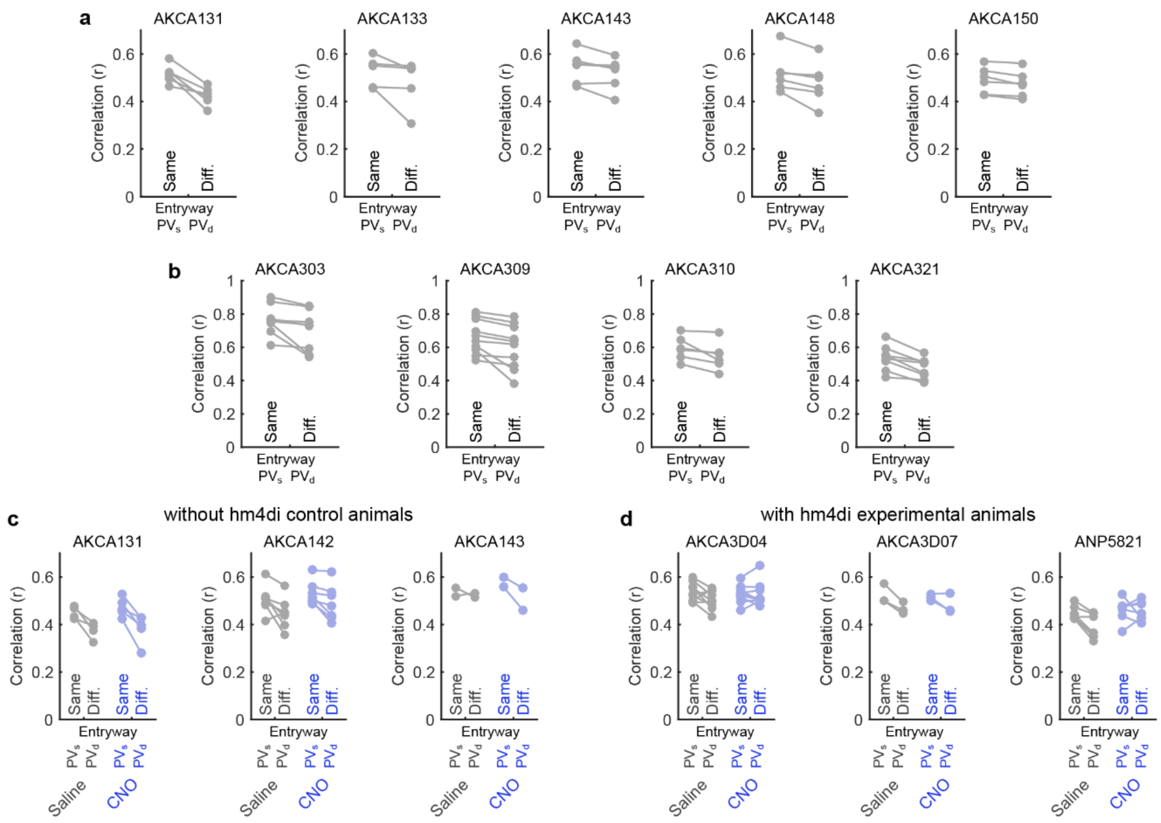

Supplementary Figure 8. Remapping of the CA1 and CA3 population codes of individual mice in all experiments. a) Split-half population vector correlations within the compartment when the mouse entered from the same versus the different entryway separated by mouse for all initial CA1 recordings. b) As in (a) except for all CA3 recordings. c) As in (a) except for all trisynaptic inhibition recordings in control mice without hm4di, separated by Saline versus CNO sessions. d) as in (a) except for all trisynaptic inhibition recordings in experimental mice with hm4di, separated by Saline versus CNO sessions. Data provided as Source Data file.

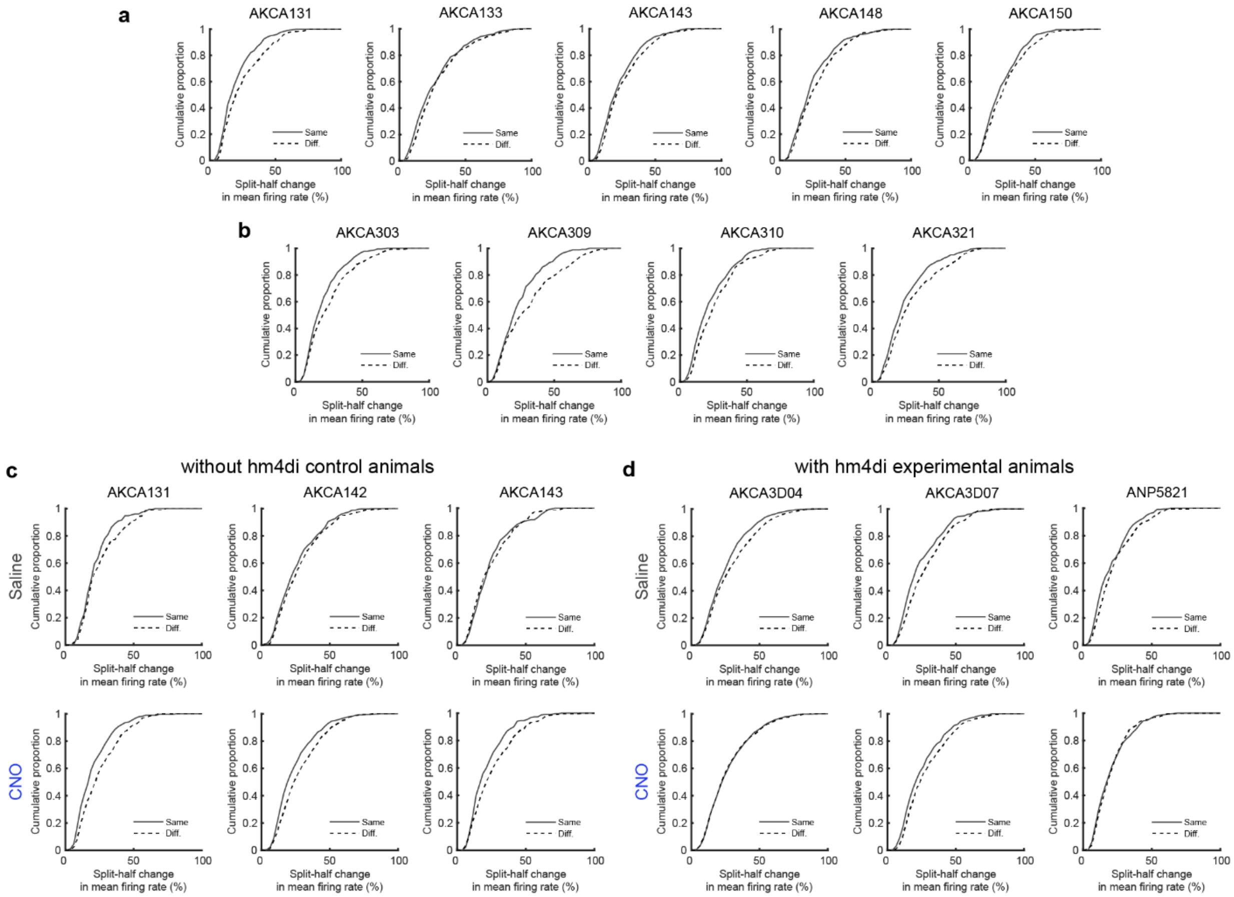

Supplementary Figure 9. Remapping of the CA1 and CA3 cellwise rate codes of individual mice in all experiments. a) Cumulative distribution of split-half changes of mean firing rates within the compartment when the mouse entered from the same versus the different entryway separated by mouse for all initial CA1 recordings. b) As in (a) except for all CA3 recordings. c) As in (a) except for all trisynaptic inhibition recordings in control mice without hm4di, separated by saline and CNO sessions. d) as in (a) except for all trisynaptic inhibition recordings in experimental mice with hm4di, separated by saline and CNO sessions. Data provided as Source Data file.

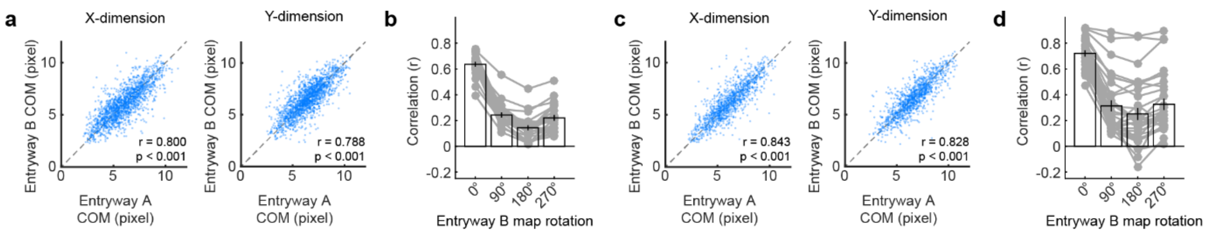

Supplementary Figure 10. Field locations and orientations are preserved during remapping of the CA1 and CA3 place codes by entryway. a) CA1 place field locations within the compartment when the mouse entered from entryway A versus entryway B were highly correlated. Place field locations were computed as the center of mass (COM) of activity across the entire compartment rate map. b) Population vector correlations of the CA1 code within the compartment when the mouse entered from entryway A versus entryway B, when the relative orientation of the entryway B map is rotated (29 sessions from 5 mice). In all cases, no rotation (0°) yielded the maximum correlation between maps. c-d) as in (a-b) except for all CA3 recordings (32 sessions from 4 mice). All bar graphs reflect mean ±1 SEM. Data provided as Source Data file.

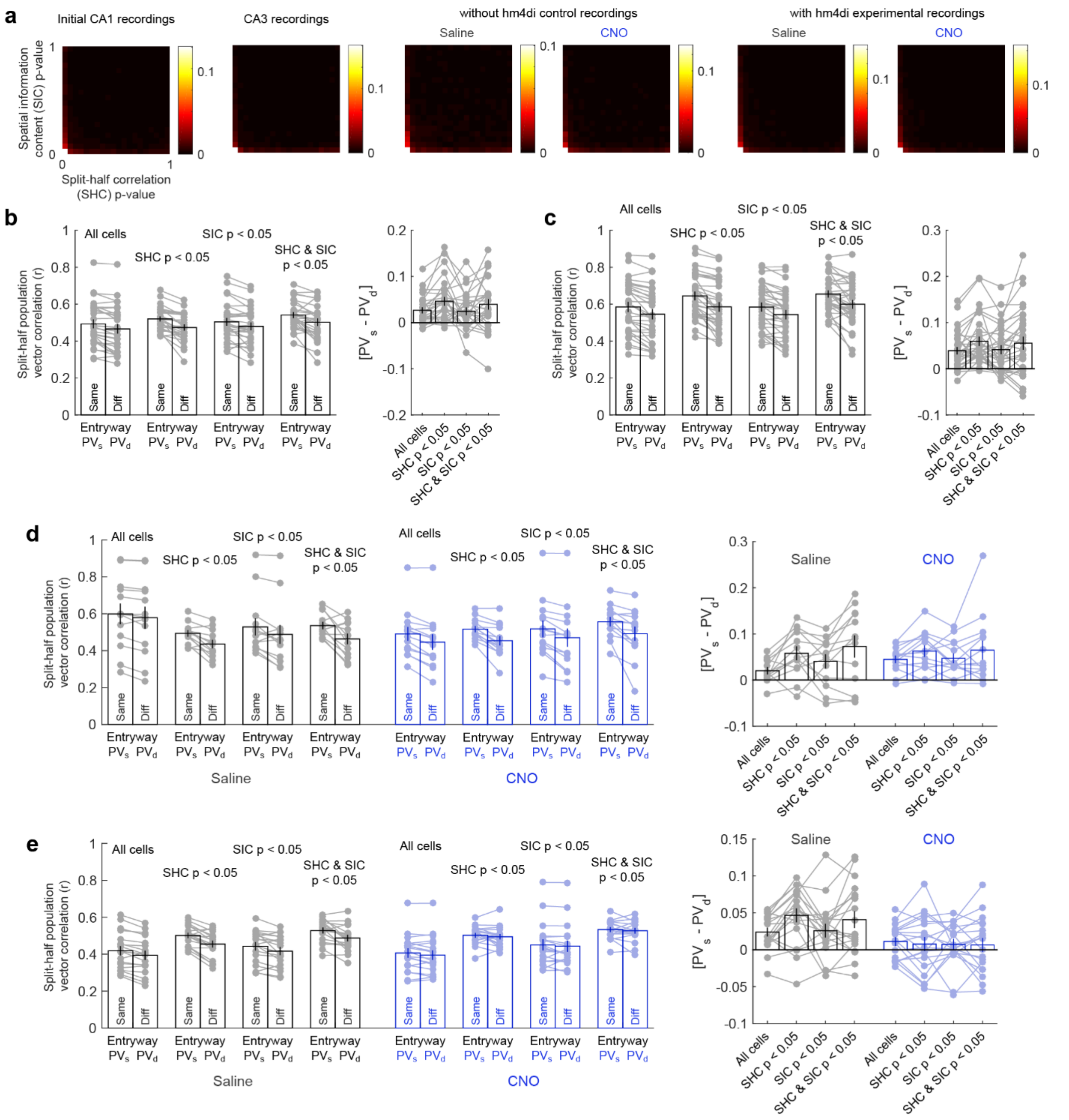

Supplementary Figure 11. Entryway remapping and DG-CA3 inhibition results are robust to a variety of place cell selection criteria. a) Joint distribution of significance values for split-half rate map correlations after matching spatial sampling (SHC) and whole-session unpartitioned spatial information content (SIC) for cells in all experiments. Cells with low SHC p-values tended to have low SIC p-values as well. Number of cells in each condition noted in **Supplementary Figure 4**. b-e) Split-half correlations of population activity within the compartment when the mouse entered from the same versus the different entryway (left) and differences in split-half correlations of population vector activity (right) when included cells are selected on the basis of various criteria. Each dot represents one session from one mouse. b) Data from initial CA1 recording sessions (29 sessions from 5 mice). Correlations were significantly higher when the mouse entered from the same entryway versus a different entryway (Wilcoxon signed rank tests: All cells: Z = 3.88, p = 1.0e-4; SHC $p < 0.05$: Z = 4.573, p

= 4.80e-6; SIC $p < 0.05$: Z = 2.80, p = 5.1e-3; SHC & SIC $p < 0.05$: Z = 2.78, p = 5.5e-3). c) Data from CA3 recording sessions (32 sessions from 4 mice). Correlations were significantly higher when the mouse entered from the same entryway versus a different entryway (Wilcoxon signed rank tests: All cells: Z = 4.39, p = 1.1e-5; SHC $p < 0.05$: Z = 4.937, p = 7.95e-7; SIC $p < 0.05$: Z = 3.78, p = 1.6e-4; SHC & SIC $p < 0.05$: Z = 3.59, p = 3.3e-4). d) Data from control mice not expressing hm4di, separated by Saline (13 sessions from 3 mice) versus CNO (14 sessions from 3 mice) sessions. Correlations were significantly higher when the mouse entered from the same entryway versus a different entryway in all conditions (Wilcoxon signed rank tests: Saline, All Cells: p = 8.1e-3; Saline, SHC $p < 0.05$: p = 4.6e-3; Saline, SIC $p < 0.05$: p = 2.7e-2; Saline, SHC & SIC $p < 0.05$: p = 4.8e-2; CNO, All Cells: p = 6.1e-4; CNO, SHC $p < 0.05$: p = 1.2e-4; CNO, SIC $p < 0.05$: p = 6.1e-4; CNO, SHC & SIC $p < 0.05$: p = 6.1e-4). Within selection criteria group, correlation differences were significantly higher under CNO versus Saline only for the all cells condition (Wilcoxon rank sum tests, Saline vs CNO: All Cells: Z = -2.26, p = 2.4e-02; SHC $p < 0.05$: Z = -0.27, p = 7.9e-01; SIC $p < 0.05$: Z = -0.12, p = 9.0e-01; SHC & SIC $p < 0.05$: Z = -0.27, p = 7.9e-01). e) Data from experimental mice expressing hm4di, separated by Saline (19 sessions from 3 mice) versus CNO (19 sessions from 3 mice) sessions. Correlations were significantly higher when the mouse entered from the same entryway versus a different entryway under Saline but not under CNO (Wilcoxon signed rank tests: Saline, All Cells: Z = 3.26, p = 1.1e-03; Saline, SHC $p < 0.05$: Z = 3.38, p = 7.2e-04; Saline, SIC $p < 0.05$: Z = 2.54, p = 1.1e-02; Saline, SHC & SIC $p < 0.05$: Z = 3.02, p = 2.5e-03; CNO, All Cells: Z = 1.97, p = 4.9e-02; CNO, SHC $p < 0.05$: Z = 0.93, p = 3.6e-01; CNO, SIC $p < 0.05$: Z = 1.17, p = 2.4e-01; CNO, SHC & SIC $p < 0.05$: Z = 0.93, p = 3.5e-01). Within selection criteria group, correlation differences were marginally or significantly lower under CNO versus Saline (Wilcoxon rank sum tests, Saline vs CNO: All Cells: Z = 1.75, p = 8.0e-02; SHC $p < 0.05$: Z = 2.89, p = 3.8e-03; SIC $p < 0.05$: Z = 1.96, p = 5.0e-02; SHC & SIC $p < 0.05$: Z = 1.75, p = 8.0e-02). Within selection criteria group, correlation differences for mice with hm4di did not differ from mice without hd4mi under Saline but were significantly lower in all CNO comparisons (Wilcoxon rank sum tests: Saline, All Cells: Z = -0.58, p = 5.6e-01; Saline, SHC $p < 0.05$: Z = 0.58, p = 5.6e-01; Saline, SIC $p < 0.05$: Z = 1.07, p = 2.8e-01; Saline, SHC & SIC $p < 0.05$: Z = 0.38, p = 7.0e-01; CNO, All Cells: Z = 3.26, p = 1.1e-03; CNO, SHC $p < 0.05$: Z = 3.33, p = 8.6e-04; CNO, SIC $p < 0.05$: Z = 2.90, p = 3.8e-03; CNO, SHC & SIC $p < 0.05$: Z = 2.50, p = 1.3e-02). All bar graphs reflect mean ±1 SEM; p-values are uncorrected and two-sided. Data provided as Source Data file.

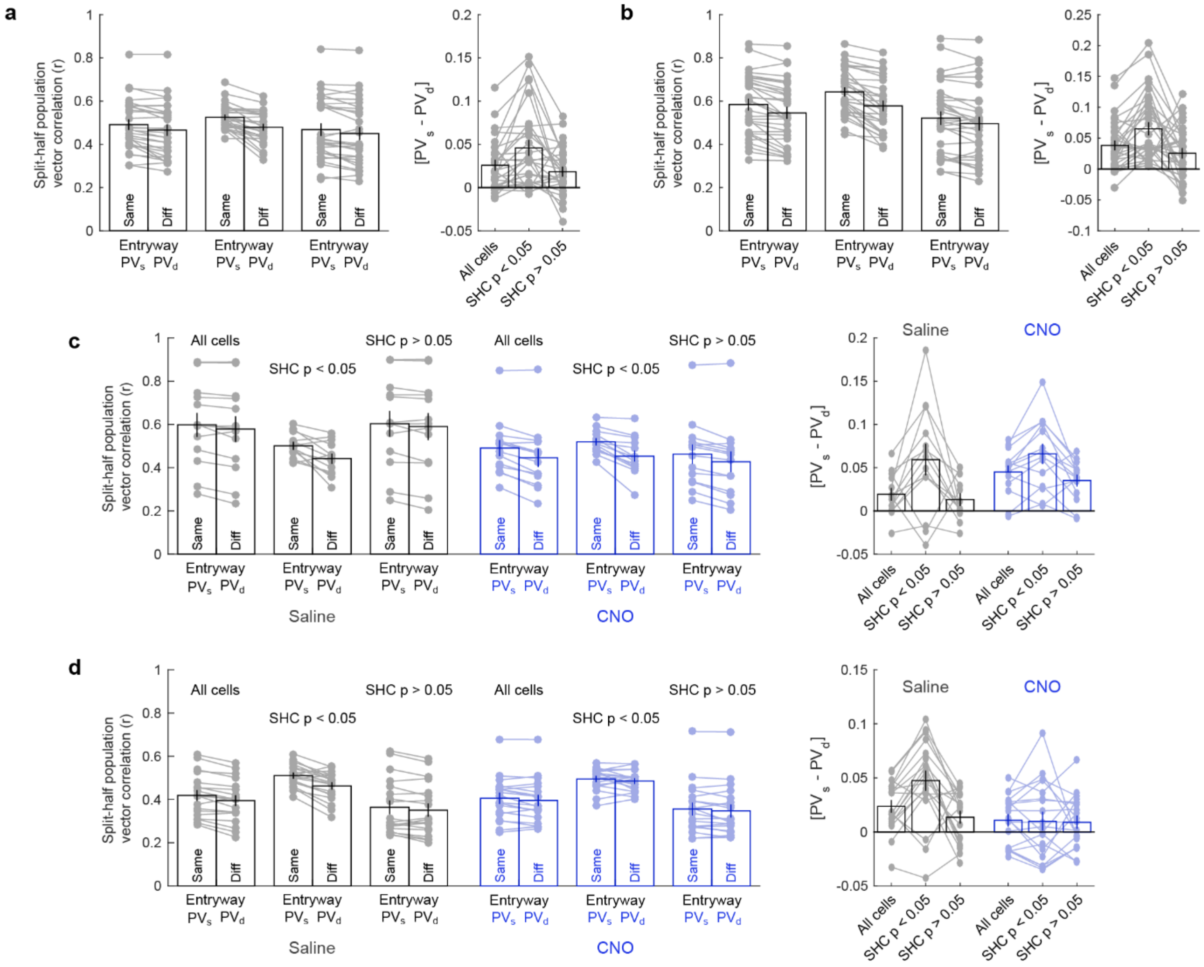

Supplementary Figure 12. Entryway remapping is more pronounced in the place cell population than in non-place cells. Split-half correlations of population activity within the compartment when the mouse entered from the same versus the different entryway (left) and differences in split-half correlations of population vector activity (right) when including all cells, cells meeting place cell criteria (split-half correlation $p < 0.05$; SHC $p < 0.05$), and cells which do not meet place cell criteria (SHC $p > 0.05$) relative to shuffled controls. a) Data from initial CA1 recordings (29 sessions from 5 mice). [PV_s - PV_d] was significantly greater when only place cells were included versus non-place cells (Wilcoxon rank sum test: $Z = 2.254$, $p = 0.0241$). b) Data from CA3 recordings (32 sessions from 4 mice). [PV_s - PV_d] was significantly greater when only place cells were included versus non-place cells (Wilcoxon rank sum test: $Z = 2.665$, $p = 0.00769$). c) Data from control mice not expressing hm4di, separated by Saline (13 sessions from 3 mice) versus CNO (14 sessions from 3 mice) sessions. [PV_s - PV_d] was significantly greater when only place cells were included versus non-place cells under Saline (Wilcoxon rank sum test: $Z = 2.000$, $p = 0.0455$) and CNO (Wilcoxon rank sum test: $Z = 2.045$, $p = 0.0409$). d) Data from experimental mice expressing hm4di, separated by Saline (19 sessions from 3 mice) versus CNO (19 sessions from 3 mice) sessions. [PV_s - PV_d] was significantly greater when only place cells were included versus non-place cells under Saline (Wilcoxon rank sum test: $Z = 3.328$, $p = 8.74e-4$) but not under CNO (Wilcoxon rank sum test: $Z = 0$, $p = 1.000$). All bar graphs reflect mean ±1 SEM; p-values are uncorrected and two-sided. Data provided as Source Data file.

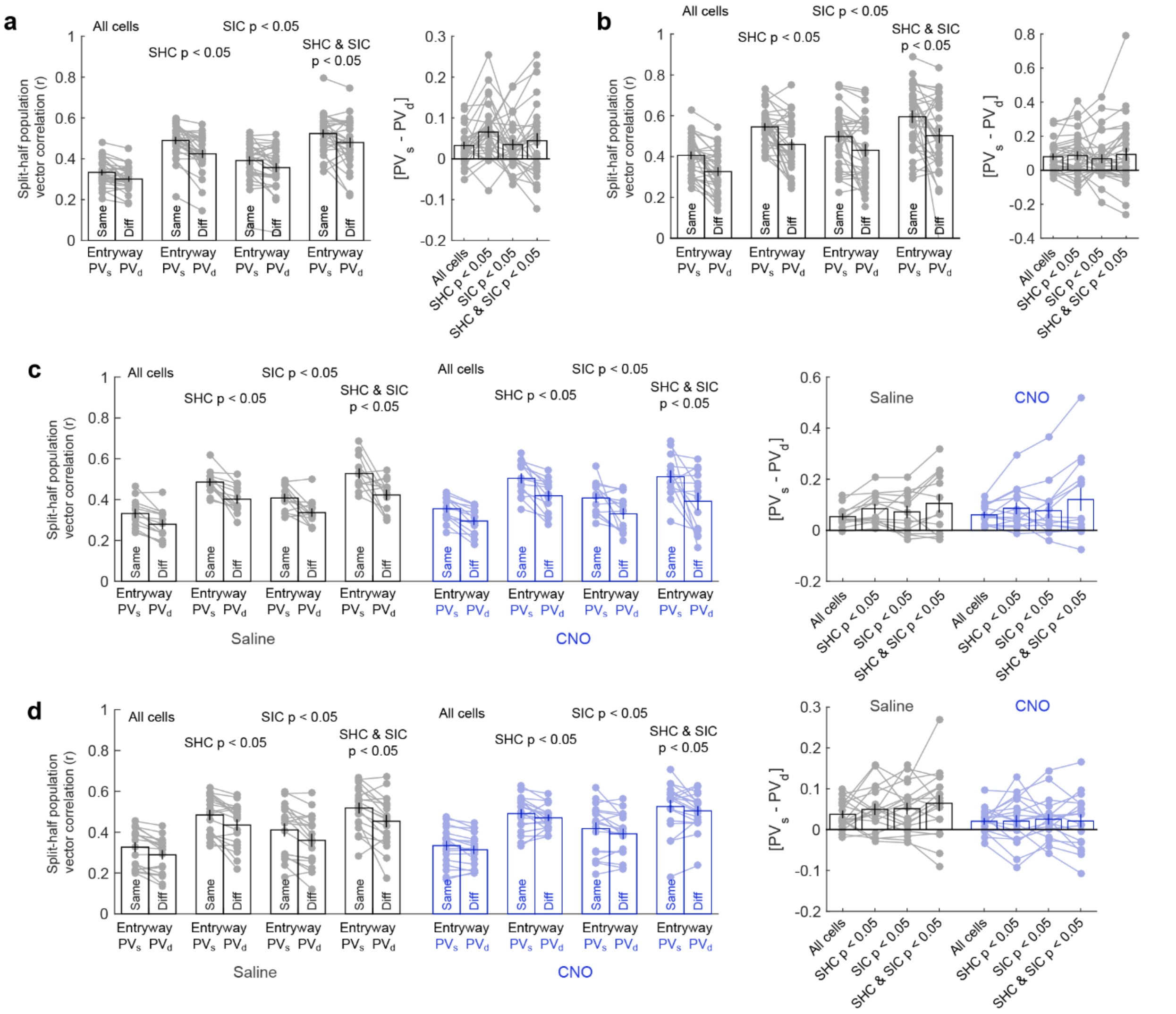

Supplementary Figure 13. Entryway remapping and DG-CA3 inhibition results are robust to other characterizations of calcium transients. To ensure that our results were robust to characterizations of calcium transients other than the estimation of firing rate via autoregressive deconvolution, here we instead binarized all dF/F traces such that periods of high-and-increasing dF/F were assigned a value of one, and all other times were assigned a value of zero (see Methods). a-d) Split-half correlations of population activity within the compartment when the mouse entered from the same versus the different entryway (left) and differences in split-half correlations of population vector activity (right) when included cells are selected on the basis of significance values for split-half rate map correlations after matching spatial sampling (SHC $p < 0.05$), whole-session unpartitioned spatial information content (SIC $p < 0.05$), neither, or both for cells in all experiments. Each dot represents one session from one mouse. a) Data from initial CA1 recording sessions (29 sessions from 5 mice). Correlations were significantly higher when the mouse entered from the same entryway versus a different entryway (Wilcoxon signed rank tests: All cells: $Z = 3.51$, $p = 4.4e-4$; SHC $p < 0.05$: $Z = 4.10$, $p = 4.2e-5$; SIC $p < 0.05$: $Z = 2.71$, $p = 6.7e-3$; SHC & SIC $p < 0.05$: $Z = 2.30$, $p = 0.021$). b) Data from CA3 recording sessions (32 sessions from 4 mice). Correlations were significantly higher when the mouse entered from the same entryway versus a different entryway (Wilcoxon signed rank tests: All cells: $Z = 4.06$, $p = 5.0e-5$; SHC $p < 0.05$: $Z = 3.42$, $p = 6.2e-4$; SIC $p < 0.05$: $Z = 3.16$, $p = 1.6e-3$; SHC & SIC p <

0.05: Z = 2.30, p = 0.021). c) Data from control mice not expressing hm4di, separated by Saline (13 sessions from 3 mice) versus CNO (14 sessions from 3 mice) sessions. Correlations were significantly higher when the mouse entered from the same entryway versus a different entryway in all conditions (Wilcoxon signed rank tests: Saline, All Cells: p = 4.9e-04; Saline, SHC p < 0.05: p = 2.4e-04; Saline, SIC p < 0.05: p = 1.7e-02; Saline, SHC & SIC p < 0.05: p = 2.1e-02; CNO, All Cells: p = 1.2e-04; CNO, SHC p < 0.05: p = 3.7e-04; CNO, SIC p < 0.05: p = 3.1e-03; CNO, SHC & SIC p < 0.05: p = 4.0e-03). Within selection criteria group, correlation differences did not differ under CNO versus Saline (Wilcoxon signed rank tests, Saline vs CNO: All Cells: Z = -0.56, p = 5.8e-01; SHC p < 0.05: Z = 0.02, p = 9.8e-01; SIC p < 0.05: Z = 0.07, p = 9.4e-01; SHC & SIC p < 0.05: Z = -0.12, p = 9.0e-01). d) Data from experimental mice expressing hm4di, separated by Saline (19 sessions from 3 mice) versus CNO (19 sessions from 3 mice) sessions. Correlations were significantly higher when the mouse entered from the same entryway versus a different entryway under Saline in all conditions and under CNO the all cells and SIC p < 0.05 conditions (Wilcoxon signed rank tests: Saline, All Cells: Z = 3.46, p = 5.4e-04; Saline, SHC p < 0.05: Z = 3.26, p = 1.1e-03; Saline, SIC p < 0.05: Z = 3.02, p = 2.5e-03; Saline, SHC & SIC p < 0.05: Z = 2.94, p = 3.3e-03; CNO, All Cells: Z = 2.54, p = 1.1e-02; CNO, SHC p < 0.05: Z = 1.41, p = 1.6e-01; CNO, SIC p < 0.05: Z = 1.97, p = 4.9e-02; CNO, SHC & SIC p < 0.05: Z = 1.17, p = 2.4e-01). Within selection criteria group, correlation differences were numerically lower under CNO versus Saline, but these differences did not reach significance (Wilcoxon rank sum tests, Saline vs CNO: All Cells: Z = 1.26, p = 2.1e-01; SHC p < 0.05: Z = 1.43, p = 1.5e-01; SIC p < 0.05: Z = 1.43, p = 1.5e-01; SHC & SIC p < 0.05: Z = 1.90, p = 5.8e-02). Within selection criteria group, correlation differences for mice with hm4di did not differ from mice without hd4mi under Saline but were significantly lower in some CNO comparisons (Wilcoxon rank sum tests: Saline, All Cells: Z = 1.15, p = 2.5e-01; Saline, SHC p < 0.05: Z = 1.19, p = 2.3e-01; Saline, SIC p < 0.05: Z = 0.58, p = 5.6e-01; Saline, SHC & SIC p < 0.05: Z = 0.54, p = 5.9e-01; CNO, All Cells: Z = 2.90, p = 3.8e-03; CNO, SHC p < 0.05: Z = 2.24, p = 2.5e-02; CNO, SIC p < 0.05: Z = 1.73, p = 8.4e-02; CNO, SHC & SIC p < 0.05: Z = 1.95, p = 5.1e-02). All bar graphs reflect mean ±1 SEM; p-values are uncorrected and two-sided. Data provided as Source Data file.

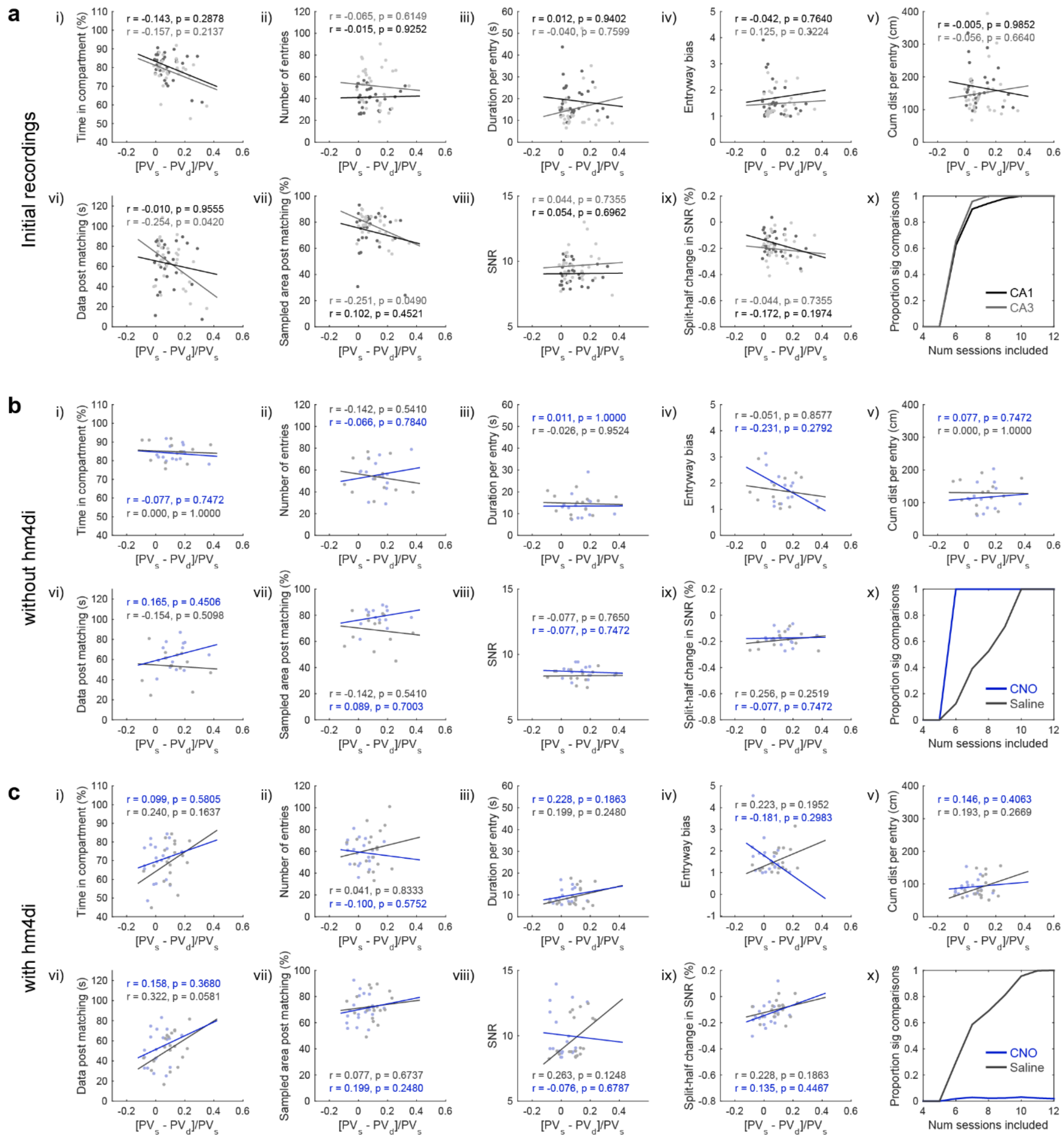

Supplementary Figure 14. Effect size is not predicted by behavioral or signal-to-noise (SNR) covariates, and effects are robust when only a subset of sessions are included. Correlation between entryway remapping and all behavioral measures described in Supplementary Table 1 (i-vii). Correlation between entryway remapping and median SNR (viii) and median split-half change in SNR (ix). Pearson's correlations for each condition reported on each plot, no comparisons are consistent across all experiments, nor do any survive correction for multiple comparisons (n = 54 comparisons, minimum observed p = 0.042). Collapsing across all experiments revealed no significant correlations (abs(r) < 0.15, ps > 0.127, uncorrected). Proportion of comparisons for which [PV_s - PV_d] significantly differed from zero when only a random subset of sessions are included (x; Wilcoxon signed-rank test, p < 0.05; 10,000 random subsets sampled without replacement for each number of sessions). a) Data from all initial CA1

and CA3 recording data. b) Data from control mice without hm4di, divided by Saline versus CNO sessions. c) Data from experimental mice with hm4di, divided by Saline versus CNO sessions. All p-values are uncorrected and two-sided. Data provided as Source Data file.

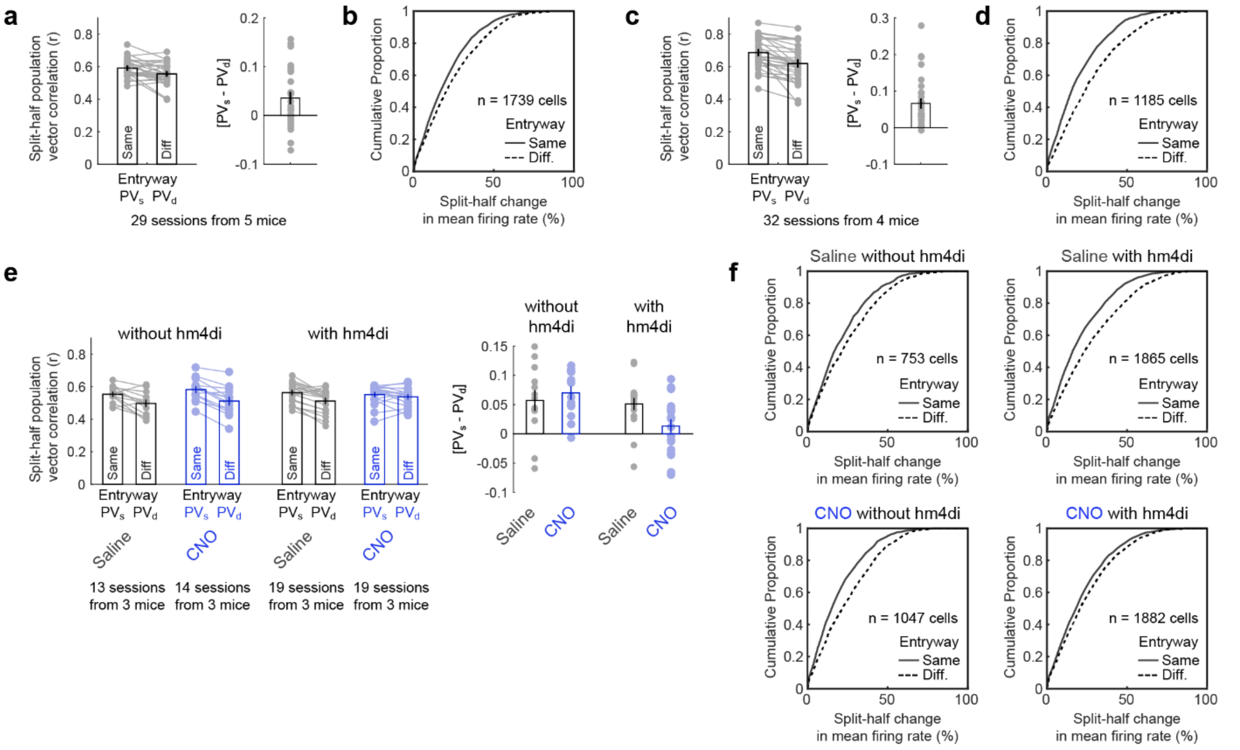

Supplementary Figure 15. Entryway remapping and effects of DG-CA3 manipulations are robust when spatial sampling distributions are no longer matched. In all analyses presented here, comparisons were computed without matching the compared spatial sampling distributions. a) Split-half correlations of population activity within the compartment when the mouse entered from the same versus the different entryway for initial CA1 recordings (29 sessions from 5 mice). Correlations were significantly higher when the mouse entered from the same entryway (Wilcoxon signed rank test: Z = 2.627, p = 8.60e-3). b) Cumulative distribution of split-half changes of mean firing rates within the compartment when the mouse entered from the same versus the different entryway for initial CA1 recordings. Mean firing rates were significantly more similar when the mouse entered from the same entryway (Wilcoxon rank sum test: Z = 5.318, p = 1.95e-7). c) as in (a) except for CA3 recordings (32 sessions from 4 mice). Correlations were significantly higher when the mouse entered from the same entryway (Wilcoxon signed rank test: Z = 4.862, p = 1.16e-6). d) as in (b) except for CA3 recordings. Mean firing rates were significantly more similar when the mouse entered from the same entryway (Wilcoxon rank sum test: Z = 8.046, p = 8.6-16). e) as in (a) except for control mice without hm4di and experimental mice with hm4di, separated by Saline versus CNO sessions. Correlations were significantly higher when the mouse entered from the same entryway in all conditions except for mice with hm4di under CNO (Wilcoxon signed rank test; with hm4di under CNO: Z = 1.288, p = 0.198; all other conditions: ps < 0.011). [PV_s - PV_d] was significantly lower for mice with hm4di under CNO than all other conditions (Wilcoxon rank sum test: Zs > 2.110, ps < 0.035; all other comparisons: Zs < 0.820, ps > 0.410). f) as in (b) except for control mice without hm4di and experimental mice with hm4di, separated by Saline versus CNO. Mean firing rates were significantly more similar when the mouse entered from the same entryway in all conditions (Wilcoxon rank sum test: all conditions: Zs > 3.291, ps < 9.951e-4). Same versus different entryway firing rate differences were significantly lower for mice with hm4di under CNO than mice with hm4di under Saline (Wilcoxon rank sum test: Z = 3.926, p = 8.65e-5) and than mice without hm4di under CNO (Wilcoxon rank sum test: Z = 2.609, p = 9.08e-3). Firing rate differences for mice with hm4di under CNO were numerically lower than those of mice without

hm4di under Saline, though this difference did not reach significance (Wilcoxon rank sum test: $Z = 0.727$, $p = 0.467$). All bar graphs reflect mean ±1 SEM; p-values are uncorrected and two-sided. Data provided as Source Data file.

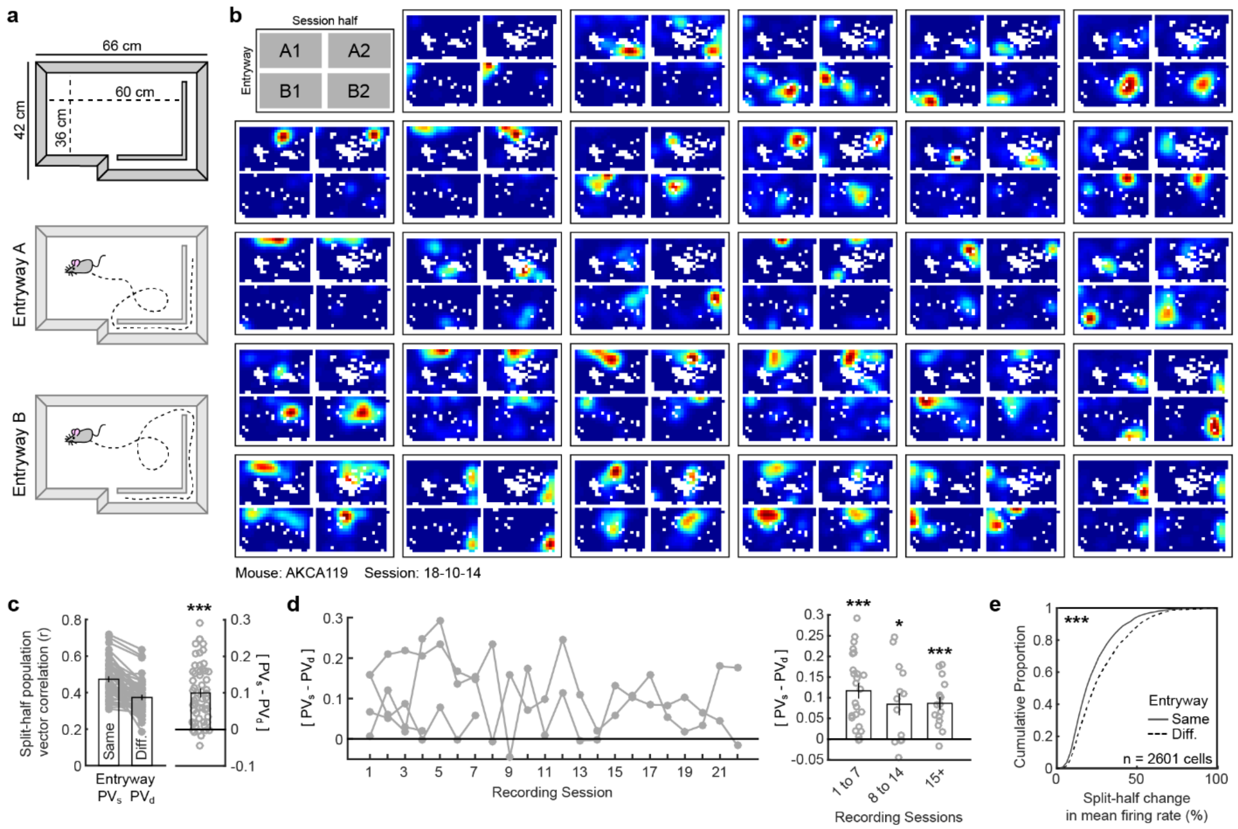

Supplementary Figure 16. The CA1 rate code depends on entryway in a larger environment. We tested whether the remapping of the CA1 rate code by entryway could also be observed in a larger environment (60 x 36 cm). To this end, we recorded 2601 place cells among 9315 cells during 55 sessions from a new cohort of 4 mice (AKCA102, AKCA110, AKCA115, and AKCA119). a) Schematic of the larger recording environment. b) Twenty-nine example rate maps from simultaneously recorded place cells in this larger environment for one session from one mouse when the data are divided by entryway and session half. Rate maps are normalized from zero (blue) to the peak (red) across all four maps. c) Split-half correlations of population activity within the compartment when the mouse entered from the same versus the different entryway (55 sessions). Correlations were significantly higher when the mouse entered from the same entryway (Wilcoxon signed rank test: Z = 6.125, p = 9.08e-10). d) Same minus different entryway split-half correlations of population activity within the compartment as a function of recording session for each mouse (left) and grouped (right; [1-7]: 25 sessions; [8-14]: 14 sessions; [15+]: 16 sessions). Correlations were significantly higher when the mouse entered from the same entryway in all groups (Wilcoxon signed rank tests: [1-7]: Z = 4.32, p = 1.5e-5; [8-14]: p = 0.0134; [15+]: Z = 3.46, p = 5.3e-4), with no reliable differences between groups (Wilcoxon rank sum tests: [1-7] versus [8-14]: Z = 1.04, p = 3.0e-01; [1-7] versus [15+]: Z = 0.79, p = 4.3e-01; [8-14] versus [15+]: Z = -0.39, p = 6.9e-01). e) Cumulative distribution of split-half changes of mean firing rates within the compartment when the mouse entered from the same versus the different entryway. Mean firing rates were significantly more similar when the mouse entered from the same entryway (Wilcoxon rank sum test: Z = 9.61, p = 6.9e-22). All bar graphs reflect mean ±1 SEM; p-values are uncorrected and two-sided. Data from (c-e) provided as Source Data file. *p < 0.05, ***p < 0.001

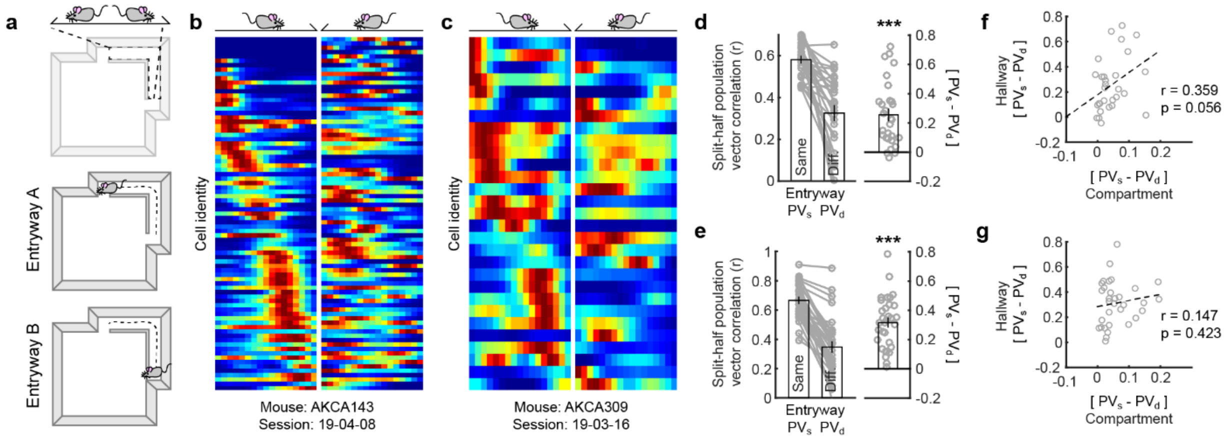

Supplementary Figure 17. The CA1 and CA3 hallway place codes depend on entryway. a) Schematic of the hallway bounds and linearization. b) Example linearized rate maps for all place cells coding for locations in the hallway partitioned by most recent entryway from the example CA1 recording depicted in Fig. 1d. Maps are normalized from zero (blue) to the peak (red) across both maps separately for each cell. c) As in (b) except for the example CA3 recording depicted in Fig. 2a. d) Split-half correlations of population activity within the hallway when the mouse entered from the same versus the different entryway for initial CA1 recordings (29 sessions from 5 mice). Correlations were significantly higher when the mouse entered from the same entryway (Wilcoxon signed rank test: $Z = 4.5301$, $p = 5.90e-6$). e) As in (d) except for CA3 recordings. Correlations were significantly higher when the mouse entered from the same entryway (Wilcoxon signed rank test: $Z = 4.9365$, $p = 7.95e-7$). f) Relationship between population vector measures of remapping within the compartment versus within the hallway for initial CA1 recordings (Pearson's correlation). g) As in (f) except for CA3 recordings (32 sessions from 4 mice). All bar graphs reflect mean ±1 SEM; p-values are uncorrected and two-sided. Data from (d-g) provided as Source Data file. ***$p < 0.001$

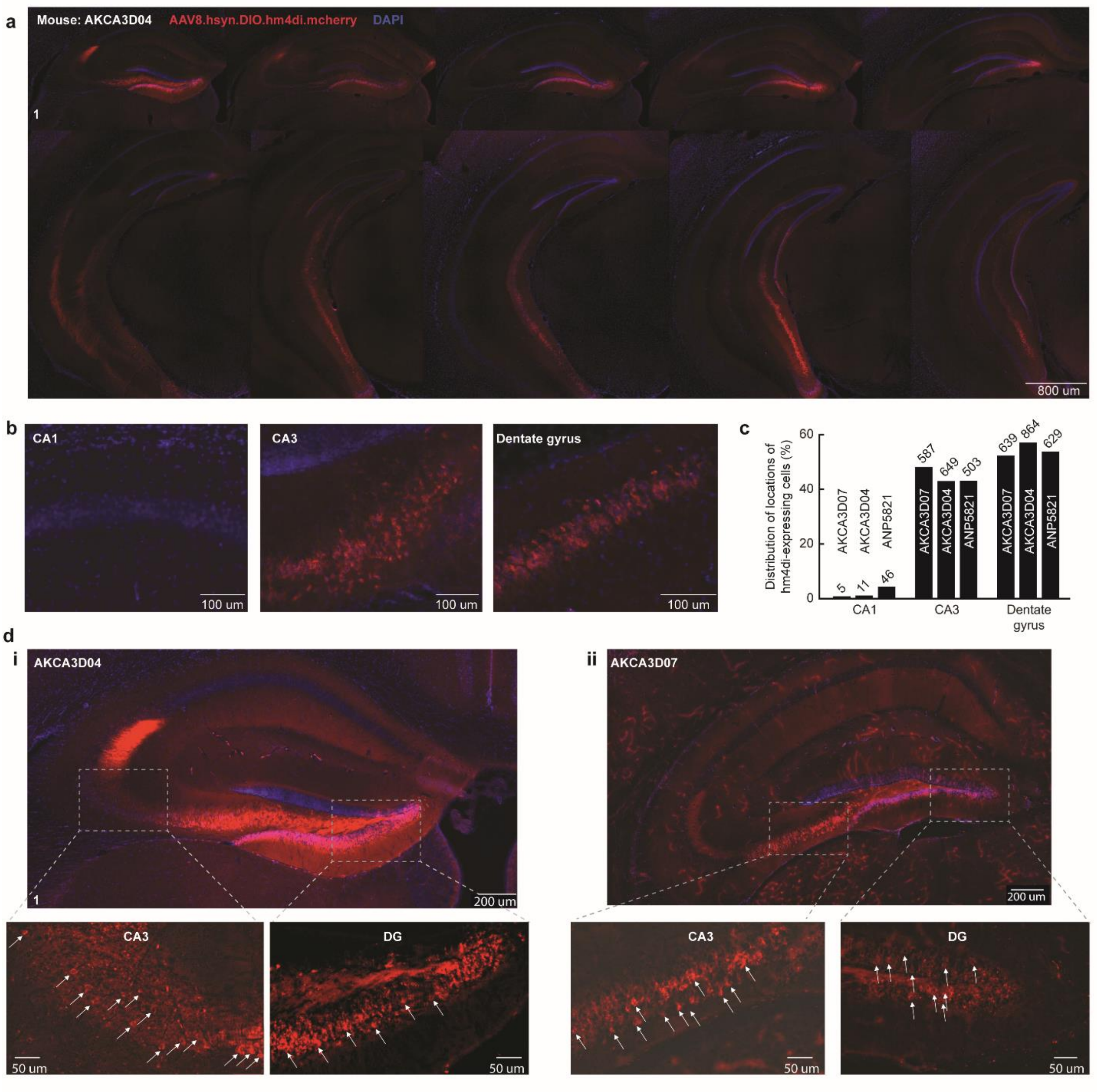

Supplementary Figure 18. Cre-mediated expression of hm4di is specific to the dentate gyrus and CA3 subregions. a) Representative examples of hm4di expression across the longitudinal hippocampal axis. Ten coronal sections taken from one of three experimental mice with hm4di. b) Representative examples of expression in specific hippocampal subregions. Single coronal section taken from one of three experimental mice with hm4di. c) Distribution of the locations of hm4di expressing cells. Raw hm4di-expressing cell counts (see Methods) noted above each bar. In all mice, expression was highly specific to the dentate gyrus and CA3 subregions. d) Representative examples of one of ten coronal sections demonstrating hm4di expression in dorsal hippocampus in two of three mice with CA3 and DG enlarged to highlight individual cell bodies (white arrows). Also note the widespread fluorescence throughout stratum radiatum in region CA1, reflecting expression of inputs arriving from region CA3.

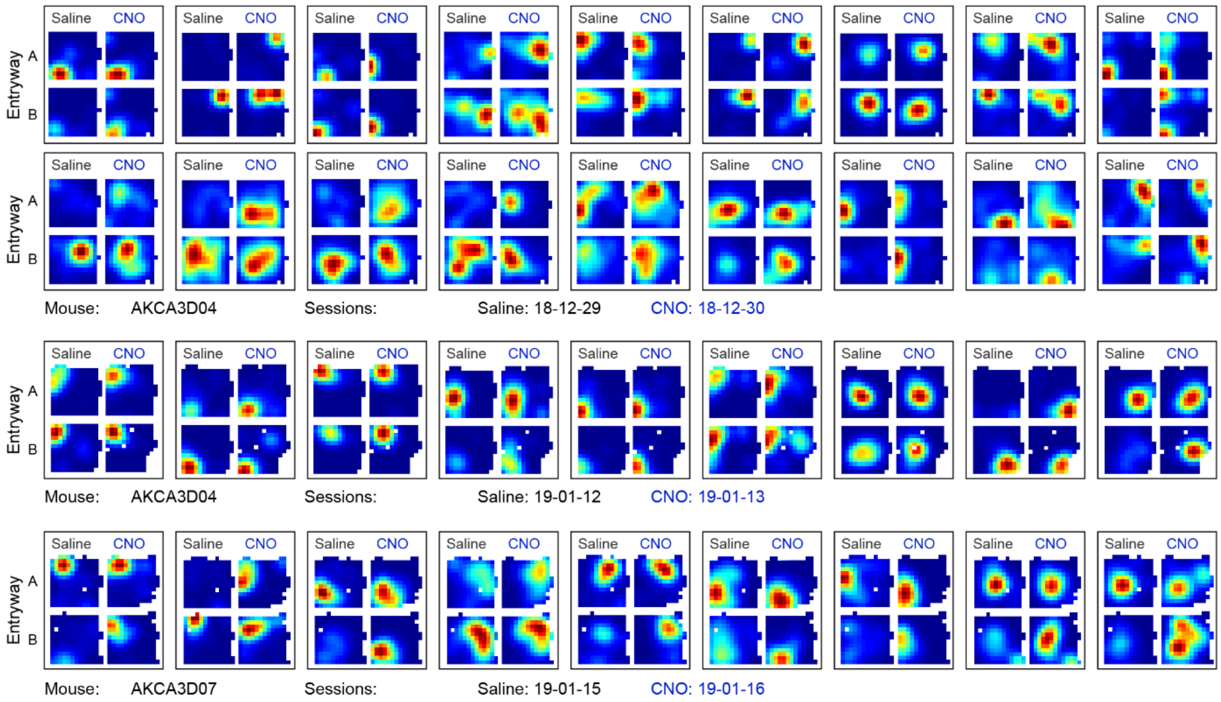

Supplementary Figure 19. Example rate maps from trisynaptic inhibition experiments for cells registered across days. The identity of individual cells was tracked across sessions using a cell registration procedure[2]. Example rate maps of simultaneously recorded cells with consistent place field locations for three sets of consecutive session pairs from two mice with hm4di. Rate maps are normalized from zero (blue) to the peak (red) across entryways within each session. Data from the third mouse with hm4di (ANP5821) could not be registered with confidence in cell identity.

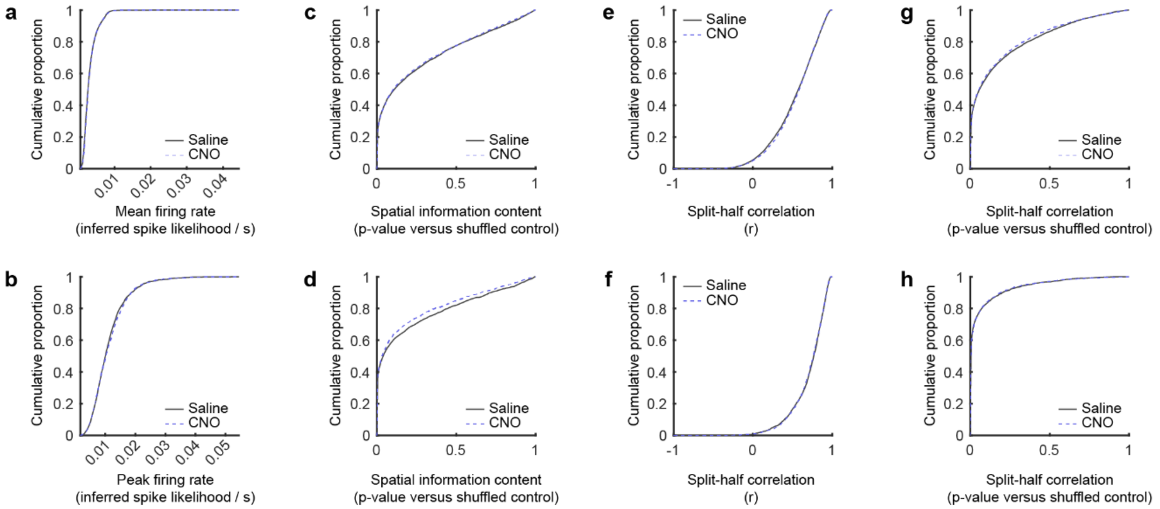

Supplementary Figure 20. Other place coding characteristics are preserved during trisynaptic inhibition. All test results listed below denote the outcome of an uncorrected two-sided Wilcoxon rank sum test between conditions. a) Cumulative distribution of mean firing rates of the whole-session unpartitioned data during Saline and CNO sessions for mice with hm4di. No reliable difference was observed (Z = 1.474, p = 0.140). b) Cumulative distribution of peak firing rates during Saline and CNO sessions for mice with hm4di. Peak firing rate defined as the maximum rate across the whole-session unpartitioned rate map. No reliable difference was observed (Z = 0.879, p = 0.379). c) Cumulative distribution of the spatial information content p-values versus the shuffled control for the whole-session unpartitioned data for all cells. No reliable difference was observed (Z = 0.542, p = 0.588). d) Cumulative distribution of the spatial information content p-values versus the shuffled control for the whole-session unpartitioned data for identified place cells. No reliable difference was observed (Z = 1.736, p = 0.082). e) Cumulative distribution of split-half correlation values for the whole-session unpartitioned data for all cells. No reliable difference was observed (Z = 1.394, p = 0.163). f) Cumulative distribution of split-half correlation values for the whole-session unpartitioned data for identified place cells. No reliable difference was observed (Z = 0.460, p = 0.646). g) Cumulative distribution of the split-half correlation p-values versus the shuffled control for the whole-session unpartitioned data for all cells. No reliable difference was observed (Z = 0.951, p = 0.342). h) Cumulative distribution of the split-half correlation p-values versus the shuffled control for the whole-session unpartitioned data for identified place cells. No reliable difference was observed (Z = 0.569, p = 0.570). Data provided as Source Data file.

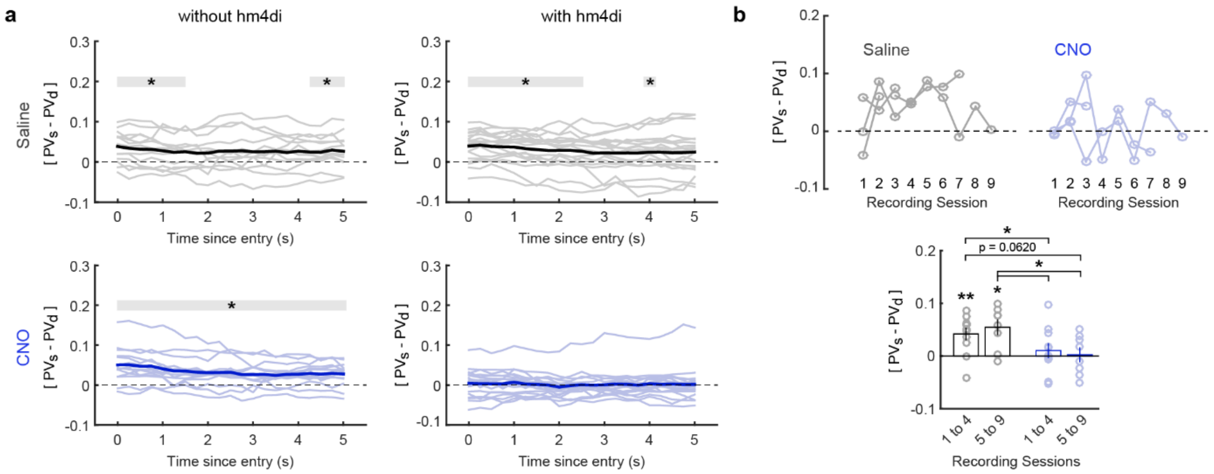

Supplementary Figure 21. Remapping by entryway during trisynaptic inhibition sessions does not emerge at later times since entering the compartment or following repeated recordings. a) [PV_s - PV_d] when only including data from progressively longer times since entering the compartment for Saline and CNO sessions for mice with and without hm4di. Computed at every 0.25 s increment. Shaded regions containing significance markers indicate all increments for which the outcome of an uncorrected Wilcoxon signed rank test versus 0 is $p < 0.05$. b) [PV_s - PV_d] ordered by recording session (top) and grouped by session number (bottom) for Saline and CNO sessions from mice with hm4di (Saline 1 to 4: 11 sessions; Saline 5 to 9: 8 sessions; CNO 1 to 4: 11 sessions; CNO 5 to 9: 8 sessions). Only Saline groups exhibited [PV_s - PV_d] greater than zero (Wilcoxon signed rank test, Saline 1 to 4: $p = 0.0098$; Saline 5 to 9: $p = 0.0234$; CNO 1 to 4: $p = 0.5771$; CNO 5 to 9: $p = 0.8438$) with the [PV_s - PV_d] of Saline groups exceeding those of CNO groups (Wilcoxon rank sum tests, Saline 1 to 4 versus CNO 1 to 4: $p = 0.0488$; Saline 1 to 4 versus CNO 5 to 9: $p = 0.0259$; Saline 5 to 9 versus CNO 1 to 4: $p = 0.0620$; Saline 5 to 9 versus CNO 5 to 9: $p = 0.0148$; All other comparisons: $p > 0.3949$). All bar graphs reflect mean ±1 SEM; p-values are uncorrected and two-sided. Data provided as Source Data file. *$p < 0.05$ **$p < 0.01$

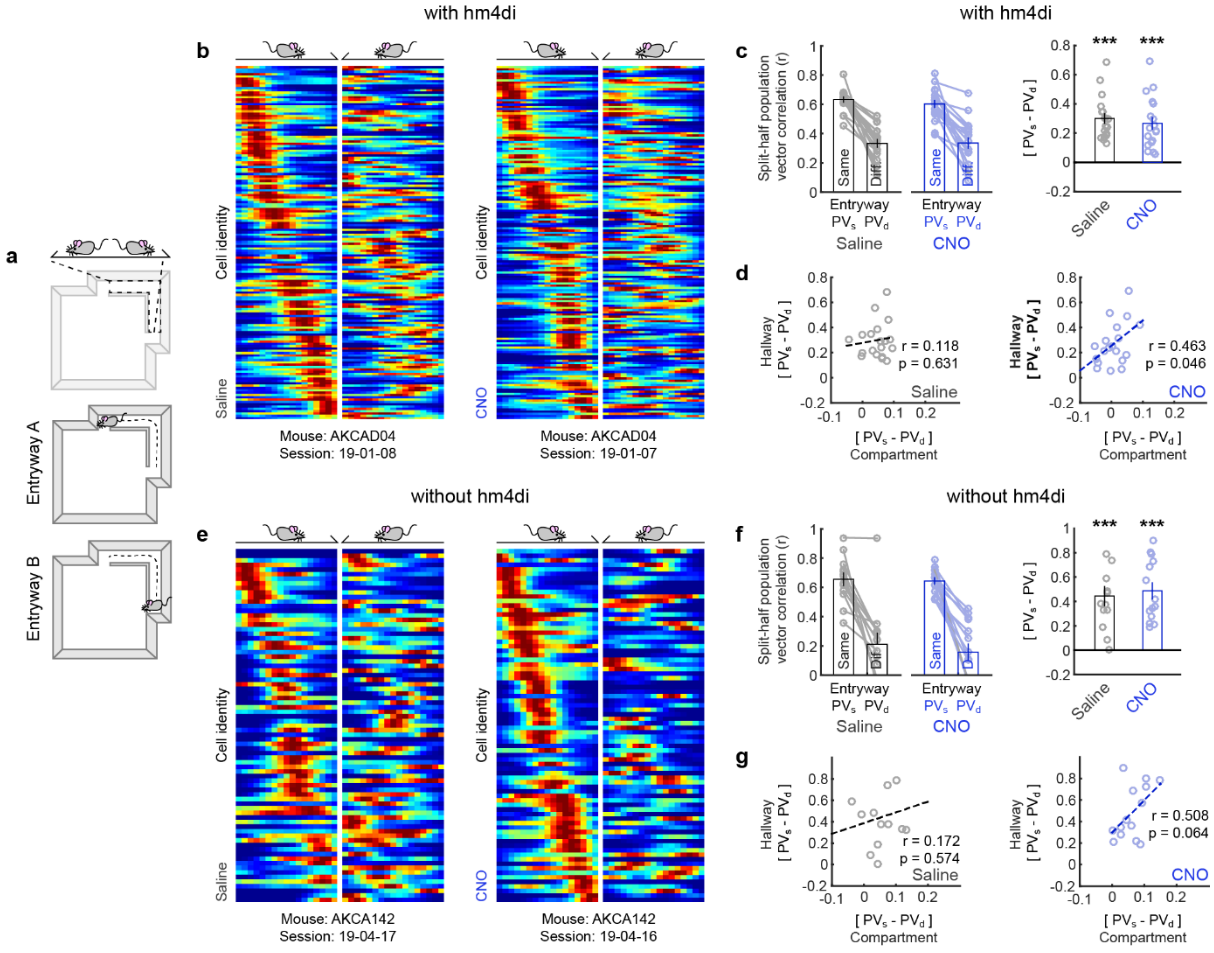

Supplementary Figure 22. Remapping of the CA1 hallway place code persists during CNO versus Saline sessions. a) Schematic of the hallway bounds and linearization. b) Example linearized rate maps for all place cells coding for locations in the hallway partitioned by most recent entryway from the example Saline (left) and CNO (right) consecutive recordings from an experimental mouse with hm4di. Maps are normalized from zero (blue) to the peak (red) across both maps separately for each cell. c) Split-half correlations of population activity within the hallway when the mouse entered from the same versus the different entryway for recordings of mice with hm4di, partitioned by Saline (13 sessions from 3 mice) versus CNO (14 sessions from 3 mice) sessions. Correlations were significantly higher when the mouse entered from the same entryway in both Saline and CNO sessions (Wilcoxon signed rank test, Saline: $Z = 3.8230$, $p = 1.32e-4$; CNO: $Z = 3.82$, $p = 1.32e-4$) with no reliable differences between conditions (Wilcoxon rank sum test: $Z = 0.7883$ $p = 0.431$). d) Relationship between population vector measures of remapping within the compartment versus within the hallway for mice with hm4di during Saline (left) and CNO (right) sessions (Pearson's correlation). e) as in (b) except for a control mouse without hm4di. f) as in (c) except for control mice without hm4di (Saline: 19 sessions from 3 mice; CNO: 19 sessions from 3 mice). Correlations were significantly higher when the mouse entered from the same entryway in both Saline and CNO sessions (Wilcoxon signed rank test, Saline: $p = 2.44e-4$; CNO: $p = 1.22e-4$) with no reliable differences between conditions (Wilcoxon rank sum test: $Z = 0.2184$ $p = 0.827$). g) as in (d) except for control mice without hm4di. All bar graphs reflect mean ±1 SEM; p-values are uncorrected and two-sided. Data from (c,d,f,g) provided as Source Data file. ***$p < 0.001$

Mouse	Experiment	Manipulation	% Time in Compartment	Number of Entries	Duration per Entry (s)	Entryway Bias	Cumulative Distance per Entry (cm)	Data post-sampling matching (s)	Area sampled post-sampling matching (%)
AKCA131	Initial CA1	No Injection	μ=84.69 σ=1.43	μ=38.17 σ=10.16	μ=22.03 σ=7.08	μ=1.57 σ=0.53	μ=190.34 σ=57.73	μ=74.66 σ=13.99	μ=78.13 σ=8.35
AKCA133	Initial CA1	No Injection	μ=74.20 σ=6.94	μ=43.80 σ=8.45	μ=16.42 σ=4.84	μ=2.65* σ=1.35	μ=143.36 σ=30.40	μ=41.03 σ=30.47	μ=59.86 σ=27.72
AKCA143	Initial CA1	No Injection	μ=86.62 σ=2.05	μ=49.50 σ=6.75	μ=16.11 σ=4.13	μ=1.35 σ=0.17	μ=148.52 σ=31.75	μ=73.14 σ=17.98	μ=79.05 σ=6.69
AKCA148	Initial CA1	No Injection	μ=71.83 σ=5.00	μ=43.00 σ=7.83	μ=15.59 σ=4.65	μ=1.48 σ=0.29	μ=140.49 σ=25.45	μ=61.46 σ=9.90	μ=78.47 σ=5.54
AKCA150	Initial CA1	No Injection	μ=84.33 σ=4.18	μ=33.00 σ=6.81	μ=25.02 σ=10.05	μ=1.64 σ=0.62	μ=215.23 σ=74.59	μ=59.76 σ=18.19	μ=67.59 σ=9.35
AKCA303	CA3	No Injection	μ=81.52 σ=1.59	μ=61.75 σ=19.75	μ=14.92 σ=8.22	μ=1.71* σ=0.54	μ=156.76 σ=63.22	μ=76.07 σ=20.03	μ=79.17 σ=9.62
AKCA309	CA3	No Injection	μ=71.32 σ=10.05	μ=59.00 σ=9.15	μ=10.78 σ=2.58	μ=1.32 σ=0.29	μ=113.65 σ=25.98	μ=47.10 σ=18.47	μ=74.72 σ=5.77
AKCA310	CA3	No Injection	μ=78.61 σ=2.19	μ=51.17 σ=11.96	μ=13.71 σ=3.92	μ=1.55 σ=0.45	μ=136.98 σ=28.12	μ=69.62 σ=8.40	μ=84.03 σ=4.07
AKCA321	CA3	No Injection	μ=82.27 σ=5.11	μ=33.50 σ=7.92	μ=23.43 σ=12.23	μ=1.43 σ=0.43	μ=200.07 σ=85.15	μ=65.17 σ=17.81	μ=74.22 σ=11.29
AKCA131	Without hm4di	CNO	μ=83.97 σ=3.91	μ=55.20 σ=12.48	μ=13.45 σ=2.22	μ=1.70 σ=0.36	μ=125.90 σ=24.53	μ=71.31 σ=11.87	μ=83.89 σ=3.21
AKCA131	Without hm4di	Saline	μ=86.53 σ=1.77	μ=48.25 σ=6.65	μ=15.31 σ=2.21	μ=1.23 σ=0.28	μ=142.52 σ=22.00	μ=54.42 σ=7.27	μ=75.35 σ=5.78
AKCA142	Without hm4di	CNO	μ=81.87 σ=1.49	μ=63.14 σ=6.64	μ=10.42 σ=2.64	μ=1.97* σ=0.69	μ=91.28 σ=22.25	μ=55.77 σ=9.31	μ=75.30 σ=7.10
AKCA142	Without hm4di	Saline	μ=82.04 σ=4.98	μ=62.43 σ=13.16	μ=12.77 σ=4.32	μ=2.12* σ=0.45	μ=108.18 σ=33.03	μ=48.97 σ=16.84	μ=65.18 σ=11.38
AKCA143	Without hm4di	CNO	μ=90.74 σ=0.96	μ=29.50 σ=0.50	μ=24.08 σ=4.99	μ=1.60 σ=0.30	μ=182.45 σ=20.12	μ=74.73 σ=3.17	μ=78.13 σ=1.74
AKCA143	Without hm4di	Saline	μ=91.46 σ=0.21	μ=35.50 σ=4.50	μ=20.13 σ=2.23	μ=1.28 σ=0.07	μ=181.34 σ=10.12	μ=67.28 σ=13.72	μ=67.71 σ=15.63
AKCA3D04	With hm4di	CNO	μ=61.85 σ=7.35	μ=56.78 σ=8.09	μ=8.28 σ=1.89	μ=1.84* σ=1.08	μ=83.70 σ=13.23	μ=44.56 σ=19.23	μ=69.37 σ=11.53
AKCA3D04	With hm4di	Saline	μ=61.06 σ=8.51	μ=58.11 σ=11.89	μ=7.54 σ=3.37	μ=1.37 σ=0.43	μ=74.64 σ=26.72	μ=39.22 σ=12.29	μ=66.28 σ=8.90
AKCA3D07	With hm4di	CNO	μ=81.33 σ=2.98	μ=46.67 σ=10.53	μ=15.01 σ=1.50	μ=1.69 σ=0.33	μ=132.09 σ=16.16	μ=58.73 σ=12.21	μ=66.90 σ=2.56
AKCA3D07	With hm4di	Saline	μ=81.99 σ=2.25	μ=45.33 σ=12.23	μ=15.19 σ=1.90	μ=1.54 σ=0.30	μ=126.51 σ=18.60	μ=67.06 σ=3.43	μ=71.06 σ=1.18
ANP5821	With hm4di	CNO	μ=75.50 σ=4.30	μ=67.43 σ=8.57	μ=8.26 σ=1.82	μ=1.55 σ=0.39	μ=80.84 σ=8.45	μ=60.30 σ=9.45	μ=73.12 σ=10.14
ANP5821	With hm4di	Saline	μ=74.18 σ=4.49	μ=74.57 σ=17.15	μ=8.90 σ=3.81	μ=1.84* σ=0.63	μ=92.63 σ=33.31	μ=61.50 σ=11.52	μ=81.15 σ=7.87

Supplementary Table 1. Behavioral characterization for each mouse for all experiments. Mean (μ) and standard deviation (σ) for median values across all sessions for each mouse. Entryway bias computed as the ratio between the number of entries through the preferred versus dispreferred entryways. Significance markers indicate an entryway bias which significantly exceeded chance relative to a shuffled control (>95th percentile; 10,000 iterations). All values computed for the entire session duration except for post-sampling matching values, which characterize the sampling within the compartment for each entryway and half after matching spatial sampling distributions. Data provided as Source Data file.

Animal ID	GCamp6f construct	GCamp6f coordinates (ML , AP, DV)	GRIN dimensions (diameter x length)	hm4di
AKCA131	AAV9.syn.GCaMP6f.WPRE.SV40	2.0, 2.1, 1.4	1.8 mm x 3 mm	No
AKCA133	AAV9.syn.GCaMP6f.WPRE.SV40	2.0, 2.1, 1.4	1.8 mm x 3 mm	No
AKCA142	AAV9.syn.GCaMP6f.WPRE.SV40	2.0, 2.1, 1.4	1.8 mm x 3 mm	No
AKCA143	AAV9.syn.GCaMP6f.WPRE.SV40	2.0, 2.1, 1.4	1.8 mm x 3 mm	No
AKCA148	AAV9.syn.GCaMP6f.WPRE.SV40	2.0, 2.1, 1.4	0.5 mm x 4 mm	No
AKCA150	AAV9.syn.GCaMP6f.WPRE.SV40	2.0, 2.1, 1.4	0.5 mm x 4 mm	No
AKCA303	AAV9.syn.GCaMP6f.WPRE.SV40	1.9, 2.0, 2.05	0.5 mm x 4 mm	No
AKCA309	AAV9.syn.GCaMP6f.WPRE.SV40	1.9, 2.0, 2.05	0.5 mm x 4 mm	No
AKCA310	AAV9.syn.GCaMP6f.WPRE.SV40	1.9, 2.0, 2.05	0.5 mm x 4 mm	No
AKCA321	AAV9.syn.GCaMP6f.WPRE.SV40	2.1, 2.3, 2.4	0.5 mm x 4 mm	No
AKCA3D04	AAV9.syn.GCaMP6f.WPRE.SV40	2.0, 2.1, 1.4	1.8 mm x 3 mm	Yes
AKCA3D07	AAV9.syn.GCaMP6f.WPRE.SV40	2.0, 2.1, 1.4	1.8 mm x 3 mm	Yes
ANP5821	AAV5.CaMKII.GCaMP6f.WPRE.SV40	2.0, 2.1, 1.4	1.8 mm x 3 mm	Yes
AKCA102	AAV9.syn.GCaMP6f.WPRE.SV40	2.0, 2.1, 1.4	1.8 mm x 3 mm	No
AKCA110	AAV5.CaMKII.GCaMP6f.WPRE.SV40	2.0, 2.1, 1.4	1.8 mm x 3 mm	No
AKCA115	AAV5.CaMKII.GCaMP6f.WPRE.SV40	2.0, 2.1, 1.4	1.8 mm x 3 mm	No
AKCA119	AAV9.syn.GCaMP6f.WPRE.SV40	2.0, 2.1, 1.4	1.8 mm x 3 mm	No

Supplementary Table 2. Calcium recording injection and implantation details for all mice included in main text and supplemental analyses. All medial-lateral (ML) and anterior-posterior (AP) coordinates indicate mm from Bregma. Dorsal-ventral (DV) coordinates indicate mm from brain surface.

ML (±)	AP	DV	Volume (nl)
1.2	-1.3	2.1	250
1.9	-1.7	2.2	500
2.4	-2.1	2.3	500
2.6	-2.3	2.5	250
3.0	-2.5	2.9	500
3.0	-2.8	2.9	250
3.0	-2.8	3.9	500

Supplementary Table 3. Injection schedule for bilateral hm4di transfection. All medial-lateral (ML) and anterior-posterior (AP) coordinates indicate mm from Bregma. Dorsal-ventral (DV) coordinates indicate mm from brain surface.

Supplementary References

1. Paxinos, G. & Franklin, K. B. J. *The mouse brain in stereotaxic coordinates*. (Academic press, 2019).
2. Sheintuch, L. *et al.* Tracking the Same Neurons across Multiple Days in Ca2+ Imaging Data. *Cell Rep.* **21**, 1102–1115 (2017).
