# Supplementary material for: DG–CA3 circuitry mediates hippocampal representations of latent information
Source: Nat Commun. 2020 Jun 15;11:3026. doi: 10.1038/s41467-020-16825-1 (PMC7296021; doi:10.1038/s41467-020-16825-1)
Supplement: Supplementary file 3 — Reporting Summary [file 41467_2020_16825_MOESM3_ESM.pdf]

nature research

Corresponding author(s): AT Keinath
MP Brandon

Last updated by author(s): May 11, 2020

Reporting Summary

Nature Research wishes to improve the reproducibility of the work that we publish. This form provides structure for consistency and transparency in reporting. For further information on Nature Research policies, see Authors & Referees and the Editorial Policy Checklist.

Statistics

For all statistical analyses, confirm that the following items are present in the figure legend, table legend, main text, or Methods section.

n/a	Confirmed	
☐	☒	The exact sample size (*n*) for each experimental group/condition, given as a discrete number and unit of measurement
☐	☒	A statement on whether measurements were taken from distinct samples or whether the same sample was measured repeatedly
☐	☒	The statistical test(s) used AND whether they are one- or two-sided<br>*Only common tests should be described solely by name; describe more complex techniques in the Methods section.*
☒	☐	A description of all covariates tested
☐	☒	A description of any assumptions or corrections, such as tests of normality and adjustment for multiple comparisons
☐	☒	A full description of the statistical parameters including central tendency (e.g. means) or other basic estimates (e.g. regression coefficient) AND variation (e.g. standard deviation) or associated estimates of uncertainty (e.g. confidence intervals)
☐	☒	For null hypothesis testing, the test statistic (e.g. *F*, *t*, *r*) with confidence intervals, effect sizes, degrees of freedom and *P* value noted<br>*Give P values as exact values whenever suitable.*
☒	☐	For Bayesian analysis, information on the choice of priors and Markov chain Monte Carlo settings
☒	☐	For hierarchical and complex designs, identification of the appropriate level for tests and full reporting of outcomes
☒	☐	Estimates of effect sizes (e.g. Cohen's *d*, Pearson's *r*), indicating how they were calculated

Our web collection on statistics for biologists contains articles on many of the points above.

Software and code

Policy information about availability of computer code

Data collection	As noted in the Methods, all calcium imaging and behavioral data collection was carried out using publicly available software downloaded from the miniscope.org site.
Data analysis	As noted in the Methods, a preprocessing pipeline of previously-reported publicly available open-source code was applied to extract neural data from the calcium imaging videos. All other analyses were conducted on these data using custom-written scripts in MATLAB (version R2015a) which are available on Github (https://github.com/akeinath/EntrywayRemapping) or via request to the corresponding authors, as noted in the Code Availability Statement. Histological images were processed with imageJ version 1.51j8.

For manuscripts utilizing custom algorithms or software that are central to the research but not yet described in published literature, software must be made available to editors/reviewers. We strongly encourage code deposition in a community repository (e.g. GitHub). See the Nature Research guidelines for submitting code & software for further information.

Data

Policy information about availability of data

All manuscripts must include a data availability statement. This statement should provide the following information, where applicable:
- Accession codes, unique identifiers, or web links for publicly available datasets
- A list of figures that have associated raw data
- A description of any restrictions on data availability

The complete dataset for all experiments are publicly available at https://doi.org/10.5061/dryad.crjdfn31g or via request to the corresponding authors. Source data for all main text and supplementary figures can be found in the accompanying Source Data Excel spreadsheet as noted in the figure captions.

Field-specific reporting

Please select the one below that is the best fit for your research. If you are not sure, read the appropriate sections before making your selection.

☒ Life sciences ☐ Behavioural & social sciences ☐ Ecological, evolutionary & environmental sciences

For a reference copy of the document with all sections, see nature.com/documents/nr-reporting-summary-flat.pdf

Life sciences study design

All studies must disclose on these points even when the disclosure is negative.

Sample size	Sample size was determined on the basis of pilot experiments in a separate cohort of mice. The sample sizes for the initial CA1 and CA3 recordings far exceeded the sample sizes necessary given the reliability of the data in this analysis.
Data exclusions	One animal was excluded prior to analysis in the initial CA1 experiments because he had a strong tendency to run through the hallway only in one direction, yielding very little data for one of the entryways. Following the decision to exclude this animal, the experimenter conducted the same analysis, which yielded results which were numerically similar to the reported animals, despite the limited data.
Replication	The primary result of rate remapping driven by entryway was initially observed in CA1 recordings (5 mice, 29 sessions) and was then successfully replicated in a larger environment (new cohort of 4 mice, 55 sessions), in CA3 (4 mice, 32 sessions), in mice without hm4di under Saline (3 mice, 12 sessions) and CNO (3 mice, 13 sessions), and in mice with hm4di under Saline (3 mice, 19 sessions). The only condition under which entryway remapping was not observed was the experimental group of mice with hm4di under CNO (3 mice, 19 sessions). All results were replicated using multiple analytic pipelines.
Randomization	Initial injection conditions were randomized, after which future sessions were interleaved.
Blinding	The experimenter carried out the injection prior to recording and therefore was not blind to the condition at the time of recording. However, all analyses were conducted blind to condition.

Reporting for specific materials, systems and methods

We require information from authors about some types of materials, experimental systems and methods used in many studies. Here, indicate whether each material, system or method listed is relevant to your study. If you are not sure if a list item applies to your research, read the appropriate section before selecting a response.

Materials & experimental systems

n/a	Involved in the study	
☒	☐	Antibodies
☒	☐	Eukaryotic cell lines
☒	☐	Palaeontology
☐	☒	Animals and other organisms
☒	☐	Human research participants
☒	☐	Clinical data

Methods

n/a	Involved in the study	
☒	☐	ChIP-seq
☒	☐	Flow cytometry
☒	☐	MRI-based neuroimaging

Animals and other organisms

Policy information about studies involving animals; ARRIVE guidelines recommended for reporting animal research

Laboratory animals	Naive male mice (Grik4-cre transgenic mouse line C57BL/6-Tg(Grik4-cre)G32-4Stl/J, The Jackson Laboratory; all other mice C57Bl/6, Charles River), aged 6-8 weeks at the time of first surgery.
Wild animals	No wild animals were used in this study.
Field-collected samples	No field-collected samples were used in this study.
Ethics oversight	All experiments were carried out in accordance with McGill University and Douglas Hospital Research Centre Animal Use and Care Committee (protocol #20157725) and in accordance with Canadian Institutes of Health Research guidelines.

Note that full information on the approval of the study protocol must also be provided in the manuscript.
